# Supplementary material for: Olfactory Sensilla and Olfactory Genes in the Parasitoid Wasp Trichogramma pretiosum Riley (Hymenoptera: Trichogrammatidae)
Source: Insects. 2021 Nov 5;12(11):998. doi: 10.3390/insects12110998 (PMC8620382; doi:10.3390/insects12110998)
Supplement: Supplementary file 1 [file insects-12-00998-s001.zip › insects-1433424-Supplementary Data S1.pdf]

>TpreOBP1
MKLKVSGSIIFILSIYLLNVQCAKMSLDELKKMVKPISSTCQKKNNVPQDLLLASYSGVFAREKSLMCYYRCLATMLKL
MNKQGQFALDKMFTQVDLLVVEELAPRIKEIAKICFDSTPKIDDPCEYTYDLVVCAYNIDSSLQGILSKSQMKNR

>TpreOBP2
MTVRPRPRLGLGLLLGCYAISLVYAGTRPSFVSDKMIETASTVVNACQIQTGVTTADIESVRDGQWPESQELKCYM
YCLWEQFGLVDEKNELSLNGMLTFFQRIPAYRNEVQNAINECKALGKYFATGDSCEYAYTFNKCYAERSPRTYYLF

>TpreOBP3
MRLSTTAMILSVFFISHIAVESKKMNIEELKKMSKPMMNSCQKKTGVKTEELEAAEKGTFPTGNKPLMCYFRCLAV
MFKLTDKDGNISLHHLLHQIDLLVIDEIAAGVNDMLQFCFEHTPKLEDSCEYIYELVICMHKRNTEMNFFEGSLLS

>TpreOBP4
MRRFASFLALFAMVVLASGDMEQMKEAFKSCKAEVGIAEDTQMKDIPSSKVGCLHACVMKKFDNMKDGKVVVE
NILQRAEKKMNPLPEEMKEKLTKCADDANGKGDECEVASYMHECWWDSMKSMGPPKGPSN

>TpreOBP5
MQKIALCLAIFLVTYRVEAANEVPAEIRDLIAGVREKCHRETGVDIEHVDRTVEGYFHPSETLGCYFSCVFNQFNLLD
HDGHLNFDEVLKRLEGLESFKEHGTEMIEKCRHLTGKNPCDSAFNLVQCFQQTNPEKFFVI

>TpreOBP6
MQPARVFSALAAILTFQALVVYAKRPEYITDEIMDMISNDKNRCMAEYGTTEALIDQVNDGHIPNDRAITCYMYC
MFESFSLVDEDGEIEIEMLVGFIPEEFQEIAAELIEACATLPGEDVCDKMYKRSSCVQAKRPDLWFMV

>TpreOBP7
MYCTNRLFIAGAQAAALGHVSPVSESFYACLAESNLTKAQFIETLKSNDTEVAQCIASCTMEKEKFMTGEQIHENAII
KKMAEVSQEIGREAITYLVKVCAEEARELKGKCGVAHSVVRCIHDSLLAEGWI

>TpreOBP8
MKNTFFIGFCIFCIVCTGEVFSAATQEQMESMSNGLRRTCVNKLGITTADIEGIRGGNFVDSPGARCYIKCVMGLM
KTFTKQGTIDIDVLVKQISIMTPSTIGKKLIEGAKTCYDEVSSDDPCELAWMFTKCTYLKGPDSFFFP

>TpreOBP9
MSVSTHLPIYVFFCSFVVLSSVANVRSAEAPKEIQGLIAGVREKCHRETGVDIEHVERTTDGHFHESEVLGCYFSCVF
NSFDLLDHDGHMDFDKLLKKLPAVESFADHGAAMVAACRHITGANPCESAFKIMQCWQSTYPDKYFVI

>TpreOBP10
MYLIVGLVLVSCILHVHANEVPMEIKNLVAGIREKCHRETGVDIEHVDRTVEGYFHESEVLGCYFSCILNSFDLLDHD
GHIDFDKLVVRLKGTDSFREHGMEMVAACRGTTGKNPCDSAFKVFQCFQKTNPAKYFVI

>TpreOBP11
MKTTLVFLAVCLAVTFASTLKDEQKAKLREFKEACIKESGVDAAVVDGIVKGGPITRGDKIDCFSACMLKKIGIMKPD
GAIDVEAARGKVKTTNADPDKANKVIDACKDLVGKDACETGGNVFSCFITKKDFPVLD

>TpreOBP12
MKSFTFGLVLVVMGVCNAAEIPAEIKGMVAGLREKCHRETGVDIEHVDRTVDGYFHPSETLGCYFSCIFNAFDVLD
KDGHVDWDKAITKLEAVESMKEHGMEMINACRTVTGKNPCDAAFNIVQCFHKTNPEKYFVI

>TpreOBP13
MKTAAFLLVVCFVAVFAEDPIKDQAVSKDLIKACLTENGFDAAQYPAGLRNAKVPENMEQKRNCYYACMMKKM
NLMKTDGALNEENLRSKFSTNLETLNKAIDTCKAQGQNDFCKLASCMMANREI

>TpreOBP14
MVRVRLALVLAFLVSATVCHNITLTNDQLDTYIKTCLTKTRISQAFYKSDDENLKRLSERQKSCFLACMFKKSGIISDD
GTVSSVTDDQEDSATNKAIKRCTKRAKGDICRLAWCLRKLEKFSLPIIVQKPRIVQY

>TPREOBP15
MKGILYLTITILCMHNVKAGEVPEEIKHLVVGLREKCHRETGVDIEHVDRTVEGYFHPSETLGCYFSCLFNSFDLLDH
DGHLDWDKAISKLDAVGSLRDHAMDFINACRGTTGANPCESALNIVQCFQKAYPDKFFVI

>TpreOBP16
MKCIFTLTCLLVLTHTIHCEYEDTMFMDEMIKCAKEMGISADQLKEALETKNDEKLSCVNACAMKHLGTLSNGKIQ
KEKIFELIDKYADKIKDSDKLKEVVTSCADEVSSSGDMPECQLARKFTTCFENHFKV

>TpreOBP17
MKLFIEIFILAVAAFCLVTAGRPDFVTDEILEMVAGDKARCMNEHGTTESMIDAVNEGNIMNDRAITCYMYCLFEA
FSLVDEDGILEVEMLVGFLPENMQASAETIVNSCIDESPGDVCDKMYATAKCIYDKRPDLWFML

>TpreOBP18
MKYLAVILAFCLAGAYAGLSDEQKAKLVEHRKVCVAETGLDPVVVENIKKGQPVQFDEKLSCFAACMLKRIGIMRP
DGSMDEQVARAKLPKDLPKDKVDAVINSCKTQVGRNQCETGGKVLGCLLKTKAVSILA

>TpreOBP19
MKIKLATCIIILINLSAIDSKMTIEQLKNTMKPFKNTCLKKVADVDPVMVEGTKQGNFPDDPTLKCFFKCTLQMLKVL
KNGELSVPAMMNQIDIMMSEELVDKTKAIVVDCDGKSKNLGDICERSFAFVKCFYEADSELYFFP

>TpreOBP20
MRLIVLFFICVLRVRADSNGSDLGSKDDDMVTCLINSGLDPGIYSGQKIGASAPTENQTNCYLACMFKKIGYMTKD
GSIDVESILSTSHGLRKRAKARQRLDEIVNQCNMHAKDDVCKLARCFQDLRKSLLEKN

>TpreOBP21
MKNIVIIISLIVTAHAADHSLDKDELEVKEYFEQCLSEHGLKESDLEELKNKADPQILCITACVFEKQGLLMKNGEFNK
KEIIKVEQEEDPNFKQDDFDEIFSFCEEKAKGIDDACLKGNTLTMCFLDEISQLDDKN

>TpreOBP22
MKFAVFTCLMVLLVVQHYPLVQCKKMNIEELKGFTKPLTKTCKTKTGISEATLAQIAKREFPPDPVLKCYFRCIAQM
GKMMDKKGNLILENMIKQVELLIVDDISPRVKSVFTECFGEMTAEESCQLAFDFIMCIERIDQELNIIV

>TpreOR1
MDSYERSTSSYYWLFRIIGIVPYKNRRFNFIPAKVISMLIIAALFNYVFVFIFGTVAKAIKGTLTARFLSRTLPLMSLWAR
LVILYSKRHYCNRTLHEFRQLWSRCADTGHENLLLLLRFNYKIRLLYIFYLANFFLFGLGAFLPTFYRRLPPLQPNGTSR
RILPFFWYTKVYDSPNYEIVYTIHCITYVCIVVSISACNVFPSFLFLVCSGFFRILRKRLNDLAKIDWSTDTSYYTSLIELVN
LHQDILSLSKRSVSLAQNLLLINLTSVTYNIAIIALEFIDPTSNRPRLLPILITLMVQLFNIQWSAECIRQEVGRLKITRKKF
KLFFICQRAKQSYLIADASYSALKLDASANSRYCIRMMIMRAQKPVEVKAGGIYSMSFESLNRISGNIYTFYTVLRNV
QT

>TpreOR2
MIKKLTEKFTKIDYDNFENYDPFLFLKFMYILCGIWPLDKSYYPNYARKIIHILPSLSMTGIFLIMMSAFFSPSVSTEDFI
EIFSLQSAAITALTRMYFLLLRKPQVYSMIYHTIEMETKGPIQLLSKEECIIINKWRIIQDFLTKMLSVGYLLGVIFYLIPP
AFFRKLPFPVFIPENLCTEWYTRLYIIECIAIFLSCPAWVSLDLYLCIFLCQLCMHLELVYAAVQDLRGKDRATLHTIIRR
HSKVLMYGQKLCEVVHYIFFAICCSYGSFLIFGTITIGEISWKTHGALAIKNIVTLVVCSSTIYLICYVGELLQDLSSRIGD
GILLDNYAEHGSNRKYLKELEIMNDRCKLSLKIEYTPNMIVNMKLFTNIMNYVVSTYIFVNTMFVNPV

>TpreOR3
MNLKIKAMSNNSAKTFIRHKQITNPAGSDGFEYIMAPCRFFLRLLGAWPDPLENDSWSSTLRILIVTTIMFLFAIVSQ
TVKLFNCWDNLNAVAEILSNCNIPTSIATVKIVNIWYHRHVLKDMLSQVIDDWKLPRTEEELTLMWQNAKVSRLLS
IGCIFMTEATLLAQCMVGLWIPIFYTFQKSQLNSSVEWPLYMTGSFPYDTQKTPYYEFTIFGQLFSNVLASTSFSSSD
SIFFILMLHLINQLSILKLSVTNLPQKIVTIQDRINFMNKFTSFHARHNQLWRFSLAVENTFNRMFLIQMVPCIFGLGT
QGYQLISNIIQEHTPLVELVFMIYFLVLFLFTIFTYCYVAELLRRQSLEISDAIFACNWRILKSREIKLLAFVMARAQKPFE
ITVGKFANFSLQLYVRILKTSAGYLSMLLAVKEKIDT

>TpreOR4
MSDIKPSSDRDKEVPKNAKLVGFEKHLFPLLFSVLGASGIWRMSRVRHPLAVLLWKLYYLLAATSVFVLFFTISAEQLI
NDEKSWREFFESLFLLLTIFNGMCKIVNVHLRRTRYLRIVSQLLQDSWLDLRDPEEVAIVEASKLDERFIVRFNMTLVF
FNNVSNAVGPLLDGNPGRDLMVDAYTPCRRSASLACYLIFYWYQVFGYNITSFVHIGCDGFFFDTVDRICAHLKILE
RRLVKLPELVGAQKFESDEEKMQFEMDYVKECVRYHHSIFEVMRELRGTVHVAIIVQLLSSVIVLCTSIYLLSCQPLFS
PEFLQLFIYFNCALFQNFFYFWFGYKITVNTLHVSQALLDMNWWTLDVKTRKMLLFVMMRTSNKVELFNSTIMILT
PESFVKILKLSYSAFNILKQTS

>TpreOR5
MNPDEVFDNKYFRLNRTLLKQVSLWPYESTSTKVIKRIFIMVGFYSMSLPQMIRGIEEIRSDDPDPEIIIENLSGFIYFH
GVISKLITQMVTENKLKYLYEEISKDWKTITDKNEKAVLEKSAAVGHQLTIFYTGFVVLSAIFFVSITAFVPVLLNHVLP
GNQTYQKQICIYAEYFVDQEKYFYYIFTHTMVIGVMTVYVATAIDSVFVNCVQHVLGLFNIIKYRLKEISRVYDSSVN
NSIDLHFDVKRYLIDIILTHKKSLEFTNLIQSAYNECFFLLAGLIVAGLSAFTYVLSQNVNNPLNFMRIWFLWFGVIVY
MFFVNLPGQKLLNISEELLLAIYDSSWHKFPIKTRFLIQVMMLRCLKPCRLTAGPLIEMNFASCSNILRTAFSYFTVVN
SMNS

>TpreOR6
MLKVHQNIDDSIFDSSAYHINKKYLIIVGAWPYLTPGKRKLMWLAINLALFTVWIPQLIHIIVIIDRKKEVIYCLMTYLC
ASMGFNSSVNGLLNNGALKKLFDSLKENWNDLQSDDERTIFATYANYGKMFTVGLSISYYLVILSYIVSALMPSMLH
YYFTGNWTIPERNIFEAELFVDPVEYYWPLFIHGIIIAFVAIWILLAYDCFFIMIVLYCCGMFSVLLHKIEQMDTELYNY
SYDQQLALEKIEEIVTYHLKCLEFAQKVEDFFCIQYVIQLLINTIVISVCGSQVINIADESPLDIFRYTYVAGSVIFRLSLTN
VCGQSVHDRSLRVYEQLIHRNWYEYPIQIRKLFIVLYNRSVEPCNLTAGKMVNLNLETFSKLMKTALSYCMMIIQTQ

>TpreOR7
MSVGVYGGIVVLVISCLPSQLINDSSSEIGLACYRSNWYQWEPKIRLAYLLVMIRTSSPCGWKGTKFFENDDAFFGYI
LLYEITVSIFFLTFISLSIGSYIFYSYLIIIRFQQISKKFETLQLDQLNTYNLELSDDKIQVDVIATIKKHDKCINYRSNWYQW
EPKVRLAYLLVMIRTSSPYEWKAGPIFVLNFEMVAFALVQFKKGFVTDALQMGFGFLSCIITLVLLCIPSQFIIDVSDK
VGHACYFNTNWYEWKPKDRLVIRRTLMRTLYPCGWKAGPSITLNLRTAAFVIKTAMSYVMTMISIYA

>TpreOR8
MLKLNKFICWPLGLWPLDGDGIFSWFRLVFAGINQLWMMCVQLAAVFLNCGDSSDKVDYIMMTVCAAMALTK
MIALRVHMAKIREVLLGSLDDWTALDESKNEQDRSLMWSYGKTGRRVILYQMINCYVSNTLIFVGSLPFLMPPMD
QSNATLGNETMVGMVRQLPLRTGCMFGNTRNGLYASLYALETLTIQTTAHGNIGSDCFIFSLMMHLCGQLELLRN
RLSTIGDGTSAESKEEEEEERKKTGREIRERVERHSQLLELASGLNATLSGVLIIQLLLNAGLNLMLCIRIILALKSGVALA
AVRPLLAFGVLMLQLFLLCYASERLCQQHEALADSIYQSKWYELPIEQRRNLNFMAARSSKPIYLMAGHFYAMNLE
NFKNILKASFSYFSILRIMFDAEEKNESTRPIFRCRLSVQACTVSISA

>TpreOR9
MAEEEMKINEVDDFFDLHYFALNKKFQIITGLWPLETGYLKYLKQGAMASVIIWNLILFSHALGTFCGTNMDYCCE
NMIAFVYSTSAIFKLIGISTTGAKFTVIYRMIARNWRNTTDKVERSILEKYALISKRLSLIYIIAFSCIATIVTQLPFIPLLLN
VIMPLNESREAIIVINTDYSITPYTKSGHLWVHYSLTGTTTAAVFIATDATFLLVVFQILAIFEVVKQRIRQAVLVAQES

SEQKSYGILIKAVQLHKDAIQFLQLTDEANSLQFFAGLGGTIFMISFGSIALLIRMDAYADLFRVVILTMGYLFQLFILC
LLGELVINASTELFNFPMLTDWHVLPIKSKKLILFFMGRTIRPSYYTAGGLYIMNVQNFASIIKTAVSYIAVVLQFR

>TpreOR10
MDIFETNYFLINKKMLSLVGLWPYDDKATKNIKRSFFIAAIGVVTILPQFFGLRMKVLLDNMRKNWIKIVDKTERAILI
QYSERGFMISVGYMSYISLALIVYISTPFIPTLLDLVIPLNESRPKMHLLKGEFWFDADEHYWQIFQIDSISCIMASTVI
MTVDSFYANCAEHCLGLFAIVKYRLSIPNKENLKKTDPRYEDVSYKWLVENIRLHSAVLEFSSMLNYQIVHTTSLSVL
VLMSLEKPLDFVRFSMLLIGLIIHLFYLCWPGQKLIDHSSGLFRDAYNSQWYDSSMRSRKLLAIFILRCSKPCILTAGGI
CNMNFENFAVILKKSMSFFTVFSNIS

>TpreOR11
MFKKLFTIIYLPEIPGLVELDKNYGETLIFLFHTTFYVTQPPINWEFAIQNLFELSFVLMMFSMYFILGNMQREIHDYT
SMTVNFWDSITDSDQKEYFEKCEYIGRKVLMILSTYVLVALSFTGTMILFSLSVNNYFLLLEMIISFFSIIFTAFLTAHFG
QIVINASDDIFYDCYFCGWYKFPSNTKPYIIMMMIRALSPCELKAGPSAVLTMNYENFSAVSYDQFHVQFNKLKPI

>TpreOR12
MVVMRLLYLSCALVQMFIYCYFGNKLTSKSEEFALGLFASNWTDLSIKSKKKILFMITRSMKSVSISKLFYMVLSLDSF
VNILRISYAVLNFMRRNLVVCQTAGLLLTRQSFDEFNETFFVVLSTGFASFKGSCDLWNRAKIIRLVDMLSEPFCLAR
CQRESAIQKTYDQMGRKIEIIMLCVVELAAVIFLFGPLINHSRELPYKVLLPYDLNDTLIFSLSYVHQTFSCILVTAGTM
TNNALIVGFMMQLCSQLDILKLRFRKASDKIKEQLEKEVASGKLSNRSSRQMEVQIIRELAQHHLHAYNLAETLCDT
FYGIIVGEFCINSLVICVSVYKLSLNAGTIPEILFNALYLVCVTGEFFVYCYFGNEITYKSTTLFEAIAAIDWNPMSKEFKK
NIIFIMSRASKPIFISCGAYVYLTLESFMAVVKLSFSVFNVLKSTL

>TpreOR13
MKKSSMHSNMQDYVWALGLNRLSLRLMGVWPADTDGNDATLSRFRVPIMISVMFFNIFLPQMYALALVLNELP
LVIDNLMTSSAALASCFKLFFLWKAKNVMNPVVESMRQDWLQTRQDWSSKIMRSAAVKGRIFAMSGYLIIVGCY
CGFAVSPFLGFNIRMISNITDYGDRHMLVQSYYPYDYSKSPIFELTFGSQLVAGIFIGMSVSVPDNYFAALVFHYSAQ
FEILGARVEQLIRDDCSLDAESHFFSENWPRLVDRHVHLMTMVSAIEQSFTFINLGQILCMSCMVCCLGFELSVSSN
VXGSINLQFTYGSDVQIFSGGEYKPSALQILTLVGTLIMMMSHTLVDCIACELLASNSSGVFHHISSGLGSNITRRKIK
DIIPMLMTSKKPRKLTAGKMFNLNLASYCSILKSIAGYISMLIAVNQR

>TpreOR14
MKFDESMINVESTGLKDYNWAVGLNRWSLRLMGIWPGEKQQYLRVPFMIGIIFFCLFLPQMYALALVMEHLPLVI
DNLLTSCAALTSCFKLLFFWQNRQVLDLVIESMRKDWLRGCKDQRTRKTMLKAASRARLITTLDYSVMASCYAGFV
VAPLLGFDLRTSNNITDYPPGGRKLLVQSYYPFDYSASPTFELAYFLQLVGSFFVGMAVSIPDDYFGALILHASAQFN
VLAARIDGLIDEVTTTRDCRGPAAVADRSDSSTINRRLGRLVDRHVHLYSMVLAIEKSFSLVLAGQIFCMSVMVCCL
GFQILSVLNNMSDKPTPLQMVTLIGTLFTMMLHTLIDCLASEALASQSTEIFYRICNSRWYELPYKSMRCLVPMML
VSKTPRQIKTGKIINMSLATYCSIIKSTAGYISVLIAVSVK

>TpreOR15
MRGKKFSRAEEKEYANLIWPMKSALRMCACWPSASPVDDTLPVKLYRILVNVCVTFVCLGGALEILAFWGQVDM
NETIECSLVVSALSMATIRLIVFNRNAREMFQVLDTMRQDWTVNYTSAEDRAVLRNRCRLSFKLAKIFISSVVITWA
LFSAMPYVEALHGHRRILPFRGYYFFDLDTASSPVYLGVYVMNFTLGAFGCSTIAAATSFSLIATIHASAKFAIVKKKFE
TIDWNEPEQVKMCVQDHQNCIKYAENVETVINILALGQFVVSTGLICFAGFQVTTMMKDHGRLMKYSSFLQAAI
MELFIFSLSGQLMQTESLEIAEHAYNSQWIGCTKSINIRMVITRSRKACTITAGKFYDMSLESFLKVLSSSFSYFTVLIT
VKDG

>TpreOR16
MADFQWALGLNRLSLRMIGVWPEANRDQRHEVSRLRSMRVPFMMLWLVLAIVLPQTYALAQVYRHLDLVVDN
LITNSAALTSVIKLFLLWKNRYVLEPAIRATEDDWSSVNGNNETTARNRFELESMTRQANRARIFTVSGYVIMFGCF

AGFVFTPLFGIGIRVVNNITDPLDGRFFPLQTYYPYDIASFPLYELTYASQLLAASFVGIAFSVPDNFFGALVFHACAQ
CEILNALLREVEQIADYCTLDLVEKEGERFHRHLRDFVRRHVQIIGFVDSIEQSFNVLVLVQVLCLSVVACFLAFGVIQ
SIRSDDVNGLAIVQVVTLVATLLNLMIHTFVYCVACELLAQHSMSFFHTVYNFNWFCLRAKHSRPLLIVSMRTRHHL
KLTAGKFFDLSLHAYMKILKNTAGYISVLLAVSST

>TpreOR17
MSEEAKKILTNEEGYSYAEQMTRCIMLPVGLWPVESDYPRFLRRFLIVGSVLVTLFMMIPLSIFICVDAPNITVKIQL
MGPNLFAMLCMSKYAGVLWKGRRIETCMRRMAEDWRNVKDPLERSMMLDYTRRARNMTKFCLALYFSGGMC
YSSLMPLLKSPEVVNNVTLYPLAYQGNFIFFNPRTRPAYDYVFALHCLGSAVRHTISCGVCGIFIWFVMHISSRVDVL
GSTIERAVDRFDNRILARIVNDQLKLYGLAIELGGIFNELSFVELVGNTILICLVGYYLMSAFVQHNYTVAYNLIITLLT
MNYNIFVLCYYGQILSDKFEELGRSVYMSNWHKLSFGDARSVVLVLGWSNRPFSLTAGKLITLSIESFAKIVKTSAAY
FNVLWNMTSNSIRA

>TpreOR18
MFVLSMQNFANKRSTNEEGYSYSEQMTRYIMIAVGLWPVDADYPRFLRSFLIVGCVLVTLFMMIPLSIFICVDAPNI
TVQIQLMAPYLFTLLCMSKYANVLWKGRRIETCMRRMAEDWGKIVDPLERSMMLDYTRRARNMTKFCLALTFSG
GMCYSTFVPLSKSPDVVDNVTLHTLAYPGNYIFFNPRTRPAYDYVFALHCLGSIVKFTTSCGVCSIFIWFVMHISSRV
DVLGTTIERAVDRFDNRILARIVNDQLKLYGLAIELGDIFNELSFVELMGNTILICLVGYYLISAFFQHNYTRAYTLTISLL
SMTYNIFVLCYYGQILMDKFEELGRSVYMSNWYKLSGSSARSVVLVLGWSNRPFRLTAGKLITLSIESFAKVVELDCL
SFDPIKYLKQLDILCNFCLPKKFGEDLCDSNMKDSPYSENVGCLFQCALEHDL

>TpreOR19
MMEHVVRRYEKYKDGILFMLIVSGLWPNYDEHPEKLKIILSICSAVTTGGATLGMVYFCISNFTNVNVLTRGLGLMI
SYFSTFLKVIVLVYHKKNILKLSKGASAQFEEDLKTPANRPFLLAYFPTFSKFYYCFRYSVALNIFMMVLKPLLALRQGK
YIRTYPVKIPFEYESGGLVHWIIYALEVAAGYYCWSVTVGVDTFFGFFTLHLVGELQLLSSRFADMKPDKNHRKIIKEL
VDRHNLLIEAQRTVQKIFGLLSVWLAITCAFIICAIIFQATETKNMTAYKAVYLLCYAFLKLVQAYSYAWYGHIITVESE
RCLNEMYNSTWPGLGDVRLMKDILFMQSQKPLIFKAKSCMIVQLDMFTKIIHTSASYFFLLQTLDEGQIENFK

>TpreOR20
MSSNAKKGRRSGATFDDYTFLNRWGLTFLGIWRLNDGRRPRTALRQLHVAALFAAMTLLLIPQWLDLYVLRGNID
ANAETFVLNVFTLTAVLKLYCFHRAGDTFEKVLLDMESNWRQVMRHHDESTKKTSRRLEGHARILLATAGKGFDYT
RRYGLLMYSTACMYFVSPFLGMQKDGLRARMYPFFGWYYFDRDSDLYYGLFYLSQVVIGIVVGTCNYSMDSIFFV
AIYHSCAQFRIIQYDVERIGADEMELDHDRATTKRKLLRLIVKHQKEIRSAERLETMFNAASMQQLLVSCIIICVIGFKL
IIALNDGGFEVLVYVAFMVIALMQIFLYCRPGDELITQSLAVGYAAWQSQWTNLGTESISKLATIIQRSQRPMRIAA
GNVYVLSLPNFTMIIKTSMSFLSLLRAIYIKSD

>TpreOR21
MEEKSSFYRGVRRVQSRVLRLAGLIPLENGKLGFVGTVCLSVYVNLAFSAVSSVYVWAFVEDCRNRRFNPDITSEM
FSFVGFHLRFLYIFGRRRKLAAMLDYCESLWTRVESHEKVHVRGFVRKVSKLSCCYSGIILTTITLYVLSSQLPQLTATS
SNETVHRVLPYPFYFDVQSSPRYEILLATQIACLLTVTQTSVCVDTSIAFLIMIACGHFRLIQVRLEQISSHIDANEREA
HRSPLRIEKTGPLSKILDNKDDDRVERETRVDGKWTGRVISRKIRQCVKYHGEILQFCTEIARLSSEIFMIELISTTYNLS
LIGILLAGNMPLTEKFKFAPVLLILTTQLFVCQYPPDLLLRESLGVSDSVYFVPPFRNDRWRIDRMLLMMLRRSQRPY
QLLAGGQIKLNIESFGNMIRGAVSFFTVLRSFN

>TpreOR22
MKPFILRAGFLADMNFETFSSIIKITENTKESGFRSTLFIEDIFQVIVSLMMMYVYPFMIWKYDRIVMTVVHMKRVL
ENNDKLGQAMVKKSYEKSNRFSLFITMLFIPITVTFAIAPLVIPILSTQFLPGNQTFQKALPLHEEFFVDEEKYFYYLFAL
QFLTIMIYCTIIEGLNTLLYSGVGFIVGELQYLQYYLEKTDSCYNDMVSECKDIAYNFLTKSLVYFIEQHQKCIKLYEILYL
LTESIMFVSMLGCSTSLIIACSGMVIFMDSDVSMALKMLVLCIATLSVILFICIPGQFLSDAGDELFTKCYCVDWHGY
PPKARILLVLILLRTMKPLQLKAGFLAVLSFETFSSVLKKSMSFVTAFRSIYATL

>TpreOR23
MFQDKGYSSYYIFNRFFLTGCGIWPYGSTCLLKFFRYFWITQQIFLMSAKIIKIFEIRYDIDTVIEAVATLFYNIAATVKY
CNGVINEKKMKFLVDKIHTDWKNVSDPMEIDILSRHSSRGKLLNILYIASVYNALLSYLLLPLSPIIMDIFMPLNESRPR
QPLLIAEFFIDENKYFYSMTTYAYMTCLYGIIPLLGTDTFYMNCVHHICGMTVILSRRIKNNINGSKKELLRTKYQRTV
DCIINHQSIIEFGDGINAMYNTSFFIIIFLNTTLLTFTGVAALIKFNEGNKYEDVVRFGMFGVAEVFHLFCNNYMGQL
VVNSGDEFRKNIFNSEWYQAPIKVRELVHFIQLRNSRPILMKAGIFPLCLPNFTVVLKSSMSYFAFLQSTRY

>TpreOR24
MDLHNYFKYNEVLMSLCSQWPYQSVVKAKIIRGFFILQHISICIPEFIRFIELRNDLDNVVTCIIPIIYNIGVFIKFLNAVR
NMNKIKNIIETIKCDYTEIIDNVEVQILHKYASFGKFLTLGYISMCALTLILYLALPLSPVIVDKLNPLEKPRPKSLIYMVQ
FYVDQEKYFYVLLLHSYITTAAGVLPILATDTFYASIAQHACGMLAILGHRLEKARYSELNHVEKSVNQYKGTILTWG
QLHNMIRYGMFTAAQMFHLLFYCYQGHSVLDKSLTINDSINKSNWYGSSIRTRKLLTMMIMRSQKPLKLTAKLFPL
TLPHFTSVLRTSMSYFTLLKSVQ

>TpreOR25
MCESVTIDWLGIKDPEEKNTMKVVCERGSRLILIHFGFVLPSLLGYVLVPVVLPYVLNPYLPENMTLKRSLCAHVELF
VDQDKYFYHILIFLIHMVILIMLIVSALDLAYTSCITYVMGKIIWIGHVFEKLGEINVSDESSSSKRKMNVYVHRTVIGLI
KRHQICLDFSQTLNDTCSPKFFISIIVLLNLLSLSGSMAVVEISYDASSATKMAVAFVMILILVLVICYPSQLLIDASNDIY
FKCYTSKWYEYPVRTRRLLILMMTRAAEPCYMTIGPTVPLHFETASTIINTAMSYVTTLVSLCAL

>TpreOR26
MEQTIQLDLETIENFYSNEYFKVCKILNCMAGLWPYKGYMDKLLRRVIVSIVILGILFVPYLRGVKKWCGKNLAACSE
DIAGTVFASGVFLKYLVTFINEKKLIQVYEEVARNHLALDDPAEQAIMAKWSMDGKIKYMGYLGYLTLAGVSFSQM
IIVPQMLDLVVPLNESRQRVTITMADFGIDSDQYFYTIYVTYCVVSVVSFFVLTSIDTMYTAIIHQILGIFNVVKYRLLN
VQTANFGLNKGNDKTGRDISSLNVISAIRLHHKSLQFIDLIESTYRYCFLILIFICVIFLSFGSITVLEAVNELYNFLRLGIVL
FGVAVHLFYLSWPGQLVIDESSDVFRATYESTWYDMPKRTQVLIDIMMMRCRQPCSLTAGGLYVMSFENYGKIIK
TTGSYITVLASFRG

>TpreOR27
MSSVSWHSEYAVQMCRYFLRPIGLWVTNENSWREKFFNKLLTVSTFSLLLFLLIPCALHTFLEEPNIAVRMKLIGPM
SFAVMAIIKYSSLTRLTKRLEKCFKSVEEDWKSSDSAERKVLHRQAKIGRLLSIFSALLMYSGSFIFYHVIMPVAAVQTF
SANLEVAHDSLDINGSSSATNEKKLRILTFPTYEAWVNLDDEIAYQFVYLMQCLSGFVMDTITVGTCSLAAVFVTHT
CAQLEIVVEMSRNYVDSRKNDKTPKTSAERLTVLVKKHCRALKFATQIKDYLNGICFVEFIGCTANICFVGYYCLTEW
ERKEPISMVTYFILVISFTLNIFIFCFIGEHLAEHCKQLDSVYMNIDWYKLPGKEVVDLLMIIAVSRRPVKLMAGSFADL
TLITFSSVMKTAFTYFNLLRTII

>TpreOR28
MEIYNREYFMHSRFFLKLFGLWPFQSGKFNKIHQIMMMLPLGTLFIPHCAKAYETRHDFHIFFVCIISLLFFMHYITK
FLYLSLTEKQFQRMLKKIDNDFSLFSDRSLQIIHDYSNSAQKFNMFYSIYLISSVITFNLGVFMPRALDLIMPLNESRPL
HPIRALRYYIDALDDSFYFVLFHGMFFDMVSIVVIVGFDTLLINCAQHACALFKIASMEIRDCTEEINQSNERVNLLTQ
RSMQKNIYHRKIVKAAIIHKHALEFFDVLESTYSLLNFFIIGISLSTVTLAEFAVNATDVMMRFALLVAGQFLNILYQNY
PGQLIKDHSLEVHAICCECQWYKDDVPDESKKLLVMIMLKSAKPSCLTAGGYFVLDLQNYLQIMKASLSYFAFLRSV
N

>TpreOR29
MAGKFESIEKFYDQPFFALNKVTFRMSGWWPFQETKKRQMIWSFVWFCIVTVVIPEVIYLIQIIKDLTKVIECFMAL
TITYGAFTMAFNAWHNNDSLKKILEHIYSNWENLRDEQELRIFTDRATISRLVNIVYALAVFYNIMIHTVSPLIPPVVD
WFMIGNWTRPEKNLLEVEYLFLDPDRYYTLIYVHGAQAGFLVVFVIVTHDTFFMTITQHSCSMFTLLGHRIRKMDE
DILKRNRRHSYAMQRVKEIIFFHRDCLRFAQLLEDTMSVMFLFQLFPSVLMISVVGAQAMIRAQAIEELIKFGFIFIS

MIVRLFFICWCAQSIMDNSLVVMSYLANTRWYEYPESSKKLFQLMFMRCSRPSYLTAGNMIKTSMSYLTMILQTQ
E

>TpreOR30
MKIPIIGKPLEISLKLTGFWNEEVNKVVLALMWSSVLTTMPFSVWVAIRMSKNPLMLFDNLSVLLAQVLLYSKLLIFS
YNRRLLRNLLREMESDFESDPQLLRYIELSGDDPRRFCKYDFFVYLGSSALFWVQIGFMYVAVPVEMREPVFKVHYP
FDYKSSPVYEVLVLTQVIQGMLQCCIQAFSESLLIALVSYVCAQIDTLFARMEEFSKMCVSDDKRNLPRLSQPVYKQ
HIKVLNVFEKLNKIYFYVTFFQVFFTTVVICLSGFVVIVISDDPTILVKFAGYYFCSCWQIFSFCLAGQRLLNKSDKISMK
MYETIWYKTSIKEIHAVAFIIKRSQKPLMLSVAKSTELSMTTFTQIMKTSFSCLSVLRARYN

>TpreOR31
MSRTGFEECAGVTQWCLTSIGYWPVIHGKRHWFFQILLPIVTPTAMILFVIVPQTENIYRFRNDFSIVMDTLAVAVV
GCVLCLWKFLGLHSNQKDLQIIIENIELDWKTANEHEQIIMWKNAKKSRIVTTIILSSTIANVSNLLMGVIMGCYYAE
ANRVGNGNIERPYYVLSHFDFDAQKSPIYELIVIGQFLGCFFASLIHTGYDGLFVFTILHFSGQLHNLRFSVENVAKDC
LRKKCTLKSLLRPLIRAHQRLDNFVMIIEKSFNQLFLGQILTSSFLICLQGYRFILLLSEVKTEVVPEISFLISYFLSIILSIFM
YCYMAEQLRIQNDELFKSIFKMEWYELLCKETKLLITFMSQSKSATRITIGKFAEFSLEFFCKILKNVAGYISMLLALRD
RFVHTDLILVE

>TpreOR32
MDIIPANFRLLQFCGIWTETDSDKNILKSIWGFSLITVIFYFTIVQIIKLYFFLEDLEELIDVMFLTVTYILLCLKILNFIMRR
QSVLHLLKMFRHDMYKACSPEEEKILKMYSKSAYNMFRIILILSQSTGVFFCLLPFVTLDPENFELPFKTYQFYDDETT
LGFSVTYVIQLVALIFGIFINVSMDTMIYGFILLTSGQYELVSYRFQESISKNDEFLLKQTITHYGMVKKTVKRIQTAFM
IVIAPLFFLSSLTLCASIFQLSQNDVFTLEFLGFTMYLSCMLCQVFLYCKYGEELKSNELEFKNNIYKSNWTSLQVNQQ
KLLTMMLILANNVEAISWKGQFTLSLDTFVWLMKTSYTAFNLIHKTSG

>TpreOR33
METLNVKENQKKLDKIARVFQWNRRLMSFLGLWPDSPNLLLFVLTFGYYSYDMFLEYMDLLVYIDRPQNVMLNL
MENMAFTEIFVRILMLRVWNRQFGELLAAAEKDFEAKSYDTDEEVAKFVPFYAKAKSFMKLLISNTAFTATSFYVKP
LLGQLGPVMDYFGANGEPNSTLIFLLPYRFYVLYELDDAPTYFWTYGSYLPFVFISGFGQSAADCLMVTLVYHLSGQ
LAVLSMRIEKIDGDAKELRRHVARHAKLLRMGKIIEEAFSAIEFGHLIGATSLVCLLGYQILVCLSIGEYSVLVSLFAFIFL
VLLVLYAHCTVGESLIAESNRVCEAYYNCNWIDMRPENARLLILCMARSQKPLRLTAGKFTDLCYQTFTDSIKTSMG
YLSVLRAVM

>TpreOR34
MERRKYRRRSYIYSLADCIYINQRKKQEKLRYCSRVFEWNELLLSVIGLYPGKFSFARFYLNLAYFTTAMGLEYLDLVLS
LGDFERVVLNLTENAAFTHIYAYTLSLWLGNREIGQLLGQVASDFAADYTDQEIATLRRYYFRTRVFIEFLFVNLFTTA
SSYFMQPFTGQMDQILGYMRGSSANSSIVYQLPYRFHAFHRVDEPASYLWTSAAYAPFVLVTFFNQSSGECCLIAL
VYHVAAQMAVLASRIRGIEPGNDCTEQLANCLRRHARLLRMGQQINKVFSAMLLVHLTGIILLVCLVGYQLLWCLA
NGEYALLPSFIVWMCLLLVSLYVHCTVGETLITESDRLHGAYYDCRWNEMPPSTARWLVLAMARSSRTLTLNAGSF
STLSLATYTSSLKASLGYLSVFRTVMQSES

>TpreOR35
MSGKFESIEEFYDQPFFSLNKFTFRMSGWWPFQETKKRQMIWSFVWFCIVTVVVPEVIYLIQIIKDLTKVIECFMAL
TITYAAFTMAFNAWHNNDSLKKILEHIHANWEDLQDEHELRIFSERAAVSRLLNVIYALMVFYNIMIHTVSPLIPPAV
DWFVSGNWSRPEKNLLEVEYLVLDPDEYYTLIYVHGAQAGFLVVFVIVTCDTFFMTITQHSCGMFMLLGYRIRKM
DEDIFEKRCSSSYATRRVKEIVFYHRDCLRFTQLLEATMSVMFLFQLFPTVIMISVVGAQAMIRAKAIEELIKFGFIFIS
MIFRLFFICWCSQNIMDSSLAVMSYLTNTRWYEYPESTKKLFTLMFMRCAKPSYLTAGNVFTLNFVTYAGMIKTSM
SYLTMILQTQESNL

>TpreOR36

MGGNKGFSYAFGFCNVTLFIVGLWPKLKKSEKKDWLSIISFIFSLCIIVIFINIAQTTKLIIIWGDFVEMINNISTANLPI
MVAVIKMFIFQYYKNVLGCLLSKAMIDWQSTKTQEQESSMYENARVVRKISMICCVLGFSSVTGHLMIRICQELDFI
PNYEKKREPMVSSYFPYDYKSSPTYEITWFFQYLGAGLATLVYSGSHCLFVGLMLHLRGQVKNLSNTFRNNRTRINK
HERQDSKLFKNFIKSVVIRHNDLYE

>TpreOR37
MRKIFHNLLQLQSIFGVWTPEIENNSTVNKLIYTVNRVLIFPMPILLIVGMILALLYNNLNEEDYLETVMMLLTVLNN
FLKAVGMRLGRKYLIKIIEITRCERMSRLHDPEEVLIEKNYSIFLRKFFNSLHIMIGATWFAWVLPILFQNKNQRVLPV
KVWTGFDGLSDQDFWWFFIPDVGFFLVAAIMALHHELVLTTVLLFTCSQFDILAHRIKNVARKATKIANSTHGYVES
CESRLIEECVTHQLLIFK

>TpreOR38
MYCIIIPVLLPVTANFILPGNHTYAKQLSAHIELFLDEEIYFYNYFVMILITCYTAQVLISSWGIIYVSSAAYLVAKFRFM
GFKLKQILILSEGEDFLEKNDEIFKHVSQLVQLHQYCINYCDVLNSITSRMFGLGTLHTIMAFVISGYVIVMEYERDKN
LALKMFFVYILCSEFLLVTSHPSQMIFNSSGDIFETCYSTDWYKYSIKTRKMIHLILIRSSRPSCMKIGPSKYSNYETSGQ
LIRLSLSYIVSLMSINEQ

>TpreOR39
MPPSSSKVAASKSENEKIPYVNRDYLLDTEYVVKVAKTLLTPIGIWPRDGDDSPRSVTIFWMRIVAVFSLMLCLLVP
HFTWTFFKAEDLRKLMKIIAAMVFSSLAVLKYWNMIFTKKDIRACLETMEDHYRLVESEEARQIMLKNAKIGRLFTV
AYLSLSYGGALPYHIIMPLLQPRVLRQSDNSSMIPLPYPSEYVFFIVEDPPLYQIVFVGQILISSIILTTNTGVYSLIACIV
MHCCCLFEVTGHKLECLLDGRSYDKRAVRPDLVKRLVDIVDYHNEAIAYADTIENCLNIVMLSEMGGCTLIICFLEYG
ILQDLEDADYLGMMTYGVLMTSIFVNVFILSFIGDKVREQSELIGNSMYSIQWMDLPNDFALKNVKFIIARANQPT
RLTAGKLFDLSLQGFCDVAKTSMAYLNFLRTLEIT

>TpreOR40
MTLTRSSKDENPGFHWAFGLNRLSLKIIGIWPNEEDEDQAPKTVWKKLIALSVPLMVAGLILGIIVPQLYALNLVWQ
DFSLVIDNLTTVCIAATSTIKLFLLWNSRKLFEPILQLASDHWRRGAQRDSSRVVMLRQARRAKLFTMSGYAIMGVC
FVGFMFTPFLGLSVRIVNNVTDDLASGSFLPFQTYYPFEFHETPVYEVVYASQVLTASFAGIGFSVPDNFFGALAFHA
CAQCQLLSRMIRELPVVTMAGDKGGEFCGRLAAFVEHHLLVIRFVNLVEKAFNMIILVQTVSLTLIICFLIFGSINSLES
DDNGAAIVQIFTLSGTALNLMIHMFIYCIASELLAEYSEGIADAVYSYDWHLLPSSYSRQLVIIMIRTEFPLRFTAGKFF
YLSLNAYLNIIKSSAGYISVLLAVQGG

>TpreOR41
MRIVNSDTNITSKFPIFKYKRHWMVINMSIEKMTHKDILNMNISEFCFKYNYWNTFMLYGMALWPKIPKKLKHAS
RLSIVITYTLLIMSGGKRFMRHYNEKQLNTTLYVDYIFQTMTAMTCLICYIASMTYDHLIYSCLGKVKDVFYEISRANA
RHRMENAYKRGVLMSTIYLVMIFGCFVGFLVSPVILPIFLNYIEHNNTHELALCIHIDLFINEEKYFLVYFLVALYLVILT
GMLCSVFNGFEIWVISVIIAIFEFIGHNLDSIIQDLRINGYKNDNSTEICNKLKISINYHQKSIKIYEMLNHNSKVVLFTI
MALLLFSLSVSGSEVVLQMKIDTATTARMAFIFFASLFLLIFISYPSQILVIASEDLRIKCYACNWYEFPPKARLLLLMM
LLRTSKPCVMTAGPGVPMNYETSSKISNMAMSFVTALISLNA

>TpreOR42
MDAVFDNPNYMISKFLLRFLGLWPTQSRLRKNLSFLAYTFIIFSLVIPIFFGMLNNKTDLVIIIEDITGILYLLSIYTKYLSF
YVFEERMIRVYNQLVKDLEDITIKEEKAILRKHARQGRFLSIIYVGYGAIATMVFNSTPYIPLILDKVFPLNETRDLLFPY
YADYYFIDAVEYHYTLFTFHGGMVICAATLGATAVDSMFVVNVKHNCGLFNIVCYRLENIGKSALSKDHALPTAHAS
DEVVYREMKSVFISHRNSIESSQIIQETLSGSFLFIFAAAMAAIAMLVFDIMLNLQKPIQIIRIGVLLVGVYLSIFYMNYI
AQQIMNDSEKVNEAACNSYWYHCSPSAQKLVQLALLRSQAPIILSAGGVFDMNLATFASMIKSSASYATVLLQMQ
QK

>TpreOR43
MTTTAATKERGPSIRYKNAFSEAKKLMSWNKYLMLPLGLWPSKPNDYIFVTFFCFFYYHLFLYHVVLLVSIRSFSLMR
IIGALMENVTMVQVFLRLYTMRRYNKEYGKILEEFGQDFSEDNYESNEEKRIFLSYNIQSKRFIRIVVISLGLTAMLYFT
KPLIRQLSTXKKNNKRKAFTYDLPYRIYFWYKIADLNIFILTYVSQIPLLYTIGFTQTAMDCLTLTLVVHLCGQLGVLSER
ISKIDFINGTSKLIRAIKRHQELISTGLMLRKIYRVCLLGHFLGAAISICTLVYQLLMSISTGQKTNLVTFFVYGFLNIFRLY
THCWAGEYLIHESINVSNAFYQCEWYKLPVEDQKKIIFCIRRSQKALSLMAGNFGHFSLVMFTSIMKSAMAYLSFLR
NFI

>TpreOR44
MTFRLCVEALIGTLVNGASTQGAQFDSQYRLEEKEKRKDRVVGAAPMEIEAEYSKLTWPMRAVMSSISYWPGRE
QEEKDLGKRSSWSRLLHKCHRGVVNASMLVLTLGGSSEIVHLGRSADINDLIECCLIVSTAYLALLRVLVFATHATSM
SRIVETMRNDWTDNYRDEADKALLRDRCLWYYKLASFYICSVIFAFASFTISPYMEIMLRKDDGPMFLPFRGYYFFN
LSSVSRTEFNGIYLLNSMAGFFACGTIAGASSFSLIAAVHGSAKFAIVQKHFESVEWTSRQQVKRCVRHHQDCIKFA
DDVEDSINILVLGQFVMSTCLLCLAGFQFTTMLRDRGRCMKYLSFLQAATTNLFLYSIAAQALQTESLEVAEAIFRSK
WIGSCSSYEIRMIIMRSRKACKITAGKFYELSLESFLKVLSSSFSYFTVLFTAKYDGV

>TpreOR45
MCEVVTIDWLGISDPEEKDTMKVVCKRGSRLIFIHFGVLLPVFACYALGPVALPYVLNPYLPENMTLQKTLCVHVEL
FIDQEKYFYYILAFSIHILTLVMLIICALDLAYTSCITYVMGKIIWIGYVFEKLCEIKTSNQNLSAKRKIDIYVHQTVIGLIKR
HQKCLEFSQTLNDACSPKFIIVMSGFMICLSLSGSMAVVEISYDAISAAKMAVSFVLILILVIVICYPSQLLIDASNEIYF
KCYCSKWYEYPVRTRKLLILMMTRAAEPCCMTIGPTMPLNFETAGVVSNDRHKISF

>TpreOR46
MNVNFAPIPFRVLKCCGLWRPLSWTSWKKMTYSGFSLVSLMIVIITALLVLIAVCQMSFNDDLFAENVFLMFALIN
ATSKAANVLLFRGRFIALLNMVASERWSKLRSDEELEIRAKCDKTIRKISVYFTTAVFVAILLRVIAPLVDLFVKGEIRLP
VNAYCPCDIRNPSCYWMLYWQQAIGTGIATLIHAAKDCLICAFLLQTCSYLEILKRRIVAIPGIIASERNCGNSGNIQE
MEKRLISGCVEDHDNIFLFSKILNSSLEVMLFGQIAVTLPNLCLSIYLLSTQNIASMDFVMTIQFFSAVVIELFFFCWYG
NQVTLNSFDVEAAISTMDWTSLTVQSQKFLLQMMVRTARPILFRVGPIMDMNIDSFLSIMKTSYSAFSVLQSTKE

>TpreOR47
MHSLVRDIIIQNVEYEILPYQFLLLTFWGIWYPQNWSLWAANIQKSYFVFISFLDIIICTEMLIFFINSFGTSNFKLINFF
FVSANITGVYKAIKLMLNRKMIREFLLTYFDADWRTPKDKIEQKIHDDINAKIKRVTLIYSVSMLGIVMMKAMSPLT
GSNSVSLPVEAWYPYKVEKTSWYWLTYLHQCILGSSAVCAHIGIDTLFMGLLLKTSYQLEVLKHRLRSLNISLLSLNGK
GSTLSIENEKMLIIECIKYHQRIYSFGKKLNDKFQDILIILVVSSLPNICINIYALSTYTSRTKIDIIATLFCTTSAFMQFFIAC
WFGNEITWNSINVRDALYDLDWTVFNLDSQKLFIFIMTRSMRPMQFKIGYLLSLNLDSFIKIIKASYSSFNILQQTTH

>TpreOR48
MNVFKSEYYKINYYYLKLLGLWPNENHSRKKFKRITVIFLIVSLLIPQYIRLFEEWGRDVDIVIELIGSIFYFTGSQMKYM
SFVRVESKMKFIFEEISRHWNTLTDAKEQKLLRENGRYGRIIALGYIIPINIILVVYITVPLAPAVLDIIDPLNESRPKAFPY
FAEYFIDDQKYYFELTIHGWIVCILSVQIYATFDTTYQLCMQHVCALFSIVENRIREANKLSWRGNSDSDHKIQMRD
RSYERMIQAVVLHKEAMRFIGLIEECYTFVYSYVVLANTLLLSLTAVDTMLNFEKGNFKQMIRLGMLYIGFSFHLLYN
MNPGQNVIDSSVHIQEAAFHTNWYDSSSKTKQLLRIIMMRSWRPCKLTANGVVTLNLETFAFVFKKSISYVAVIGS
VR

>TpreOR49
MLNDQNSVDSFYDSTTYHLNKKCLILAGGWPFLNPRKRKTIWLAINFSLFIGFVAEIIYIGEIIDRTTEVINCLMVIFCAI
LAFCLSINGAVKSHSMKRLLDSVRENWNDLQSDDERNIFSKYANYGKIFTIGLSVCYYLVLLCQCVSPLIPPMLHYCIT
GNWTDPERNILDVEFFIDPVKYYWPIFIHGATSTVAAVWMLVAYDLFFVVVVLHCCGMFAVLRYKIKQMDIMLYR
YSYDQQLVLKKIEEIVMYHLKCLEFAQKIEDFFCIQCIIQILVNTIVISVCGSQVIQLADTSPQESFKFAFVGVSGTFRLA
FFNVCGQIVNDQSLRVHDQLIYTTWYEYPKRARKLFVILYNRSAEPCNLTGAKMVNLNLATYSMLMKTAMSYFM
MIIETQ

>TpreOR50
MSDDNLAGYEESISAIRFIARINGLWPFDKGARQYQCAVPACLIIFFIIIPQTTKAIYSRNDLDTVVEVLSTCELIEIVVLL
KLFGLLYNKRDFQKLLTQVEDDWKISFEYEQKIMWSNARFSKLAAIFCVIATAGSVILHSFLFLLSVNSINKSEYNNST
PVIYQLFIKSHFPFETQNSPIYEIICFSQFSAAFLSTFVFSTFDGYFFLSILHFSGQLYNLKYNVYNLITQDSIQNKSFTRKL
AVVVCRHRHIMSYIDLIENNFNLIFLLQIFSSTVVLCMQGYQFVLIISQGTRLFTSIVFIIFFMCSSIISIFVYCYIAEIIRTES
DNLLYAVYEIDWINLKSRDANLLLIFMSRLILPVKITVGKLVPFSLEYFTTVMKTSAGYLSVLLAVAKD

>TpreOR51
MCEVVTIDWLGISDPEEKDTMKVVCKRGSRLIFIHFGVLLPVFACYALGPVALPYVLNPYLPENMTLQKTLCVHVEL
FIDQEKYFYYILAFSIHILTLVMLIICALDLAYTSCITYVMGKIIWIGYVFEKLCEIKTSNQNLSAKRKIDIYVHQTVIGLIKR
HQKCLEFSQTLNDACSPKFIIVMSGFMICLSLSGSMAVVEISYDAISAAKMAVSFVLILILVIVICYPSQLLIDASNEIYF
KCYCSKWYEYPVRTRKLLILMMTRAAEPCCMTIGPTMPLNFETAGVVSNDRHKISF

>TpreOR52
MNDNEKGFDECVGVVRAIMGLIGLWPMKEWKGFQKFQTIGVLLILFLCSLIPQVTQLLIGDNDFNTVIEILVIMVLIE
IVTIVKLFALWYNNEGMKHIAIQIWKDWQVSTESEMHVMKSNARKTKFISIFCISSSTASAMSYATQFFVAVYTDEI
YVNATHLRPYFLQAYFPFGSHYSPMYEIICCWQVIAALVSCLVFSSFDGFFIFSILHFSGQLRILSKRMRNLVEEYHSEK
SLFPKLLESVIRKHLFIIRNTDSIEHNFNKIFLTQIVITSFLICLQWYQLATILTDNGPIDVGNLIFIICFVVGNMFSIFMLY
FIAQKIHNESKRLLHSVYEMTWYELSPKYSRLLLILMNRLSLPIQITVGKFAPFSLEYFAILVKTCAGYFSVLIAVKNKLN
EN

>TpreOR53
MANVKREKGFKVAMGIAESIMRFSGIWPGVEKPKINYTRFTFIPVMLMILVFVNIPQTIQLFYFDGNLSAILNVLTM
ADVPISIALVKFLVTSYNHQTLNKLLVLLNDDWKHVRDASDIEIMWQKAKTSRKVSKICMVLSAGTVFAYSGRMLY
VLYISTLTNSEDNVDKQIVRPLYFSAKFFYDTQKTPNFEITWILQMIAAFLSALAFGSIDCLFISFILHLCGQLVILQRAFE
KIGSDDVLTDANFDYTIAKLIKKHNRINESVHYIETSFNKSILFQALSSSILFCFQGYLFIIILSTAKDTEALIEVTFMLYFTT
CFMFSVFIYCYVAEFLVDESLKLNYSIFYCKWYNLPVKKSRLLIMCLLRVRKPLQVSAGKYVFLSLNLFCHIVRTSAGYI
SVLLAVREKLFIQ

>TpreOR54
MPDQADYSFAIGPCRTGLRIFGSWPDPIIPLTQLDIFRSIIVSLTILIFGFIPQLSMAIIVANARDWNGVIEILTTATVPF
VVSLTKFNVSCYQRNVLKTLTTMMKDDWNDYHLDADLKFMQENAGLGRKISQICLFLALSVVIPHCLLTTVIYFVN
WGEYGDLCLISYFPFETNRRPNYEIILVGQCFSLIFGASTHAIIDGFFSILVLHICSQFKILQRQLSQLIENCRDAQHEKS
FFEMLPKIIDRHDQLNRFVSLIDDSFNLMFLAQIMATATSLCFQGYQLVMVTSASENGISVLELTQLVFFIGSYSSSLF
VYCYVVEKLNYESYQLVNTIFSSGWYDLPSSVTKNLMLLMCRAQKPLEVTAGKFCYFSLEFYCRILKSTGGYISMLLA
VRDRLAEENQ

>TpreOR55
MIFNLNALGDFLHILEDDWKRYKTAKAEKELKLMEENAKTGRFIGLMFASFMYTAGLFFNTFIPIATMRAINRNYQT
ETLRQSNDNITHVLPIPAKVLIYTVHSSVMTSSSPKYELLFLAQYFFAFLRYTIMVGICSIMAAYVLHVCGQLDIVIMLL
NQYIDNTNTDKTIHLDCTQRKKLSIIVTCHARALRLAARIEKTFNFMNLVDFIGCTFQICFTGFLLVMTLGGKPLIWVT
WALLLISFVFNIFIICHIGEYLTQKCQEIGEIAYSIKWYDFSSKRAMNLMNIMIISSSYPTRLTAGKMVYLTMTTFSQVL
SKDY

>TpreOR56
MINADLEQELMNSLLTDPRVAVNLSKKEIGDIVNKFMEENYCWVKKTLQMAFLWPYDSAKRKYAGRFFILFNIVH
AFLSNTWTVTHKTFRKPIDFEFLIANLFEWMVVLVVLMAYTLMILKGLDMQIFFKLSTLDMVNIIEQDERIELEKCIR
RQYNLSRIFGVLIAVSFVGFAIMPGLFPVIGEFLIPYLDGNFTFKKTLCTTADLILDRDDYFYLWYFYTWVYMAVQAT
VVISIAMSFLYIVSYTEGKFNIVGLGLHKLYEFTSADNFVNNKKVDDKIHEMMREVHINHFRCIEYCNHVNRISRSLLF

ILWSLILASLSFTGSMSVILIHSNLIVFLEMSVSFGCAVSLGFFISYSGQIVTDASGSLYEKAYNCGWYHFPKRSIVYVQL
MLVRCIRPCELNAGPSVIRLNYESFSVIMNTAMSYITVMVSLV

>TpreOR57
MAKDWHRISNEEEKKILLENVKFARSMTIFCLIFMFGGGITYNTFLPYSKGEYLVDNVTHRYLAYPSYVGFFNPNVQ
EFSNEIYLVEFGGSTIVICLLGYYLIVDIERMDTFGLLTHSCLFVSITFNIFIFCYVGEILTDKCNKIGQAVFESSWSELPPA
DVKKFILVLAVAQKPVFLTAGKMITLSIRSFTNVLKASATYLNMLRTLLVNNN

>TpreOR58
MLVLPFEIWFSIEHMNDIDKLMECFGILTGILGYGSKLLMLRLSWRSVSSLVQIIISDYEATREPALRAALLKNYKIGTG
VTKLLFGTYTSLAFFIPIENCIRIARNGYTPSLLYSVPTSYPMIKKTAKNHIMVNFVQLTQVIVAGSGHALCDVFFTVLV
MHAAFKSLVLRTAIKRYFEVCTRQKKDLKVISDAMNRVILYHREFLKFTQIFEDAYCYVVFCQIFCITLQTVGSGYILILL
AEDQKMWSVEFLKYINFIMVDVFSIMAYCTAGEYYTHQSIKIYEQLCKSSWYDLSISEMKSVLFLMIHTQKFVAITSG
KFKNLQLDCCTSIMKGTYSYLSMFRATRNRD

>TpreOR59
MKRIDYQFDLRVFTIEETKKDWICSELKFIIHHHHNLLQLHKLAQKLLSSIFLVLLMGIAFDLVLIGTWMVILLDKEVIE
ALKMIMAFLIIQFTVFTMCIPSQLMSNASYDLFVQNYSTNWYKYPPQARRMLVLLIAGTTRNFEIKAGPIFIMTFETC
SSLIKLFQELKKDNVDSNTVVECALQISVYFRHLQGYIFISTNFDMVLKFLAIMNHIFQNNSKLGQKIIMHRVDKALN
LSTLQSVAFILMCISYVITPFTFQTFLSWIFPEHYMKRIDYQFDLRVFTIEETKKDWICAELKFIIHHHHNLLQLHKLAQ
KLLSSIFLVLLMGIAFDLVLIGTWMVILLDKEVIEALKMIMAFLIIQFTVFTMCIPSQLMSNASYDLFVQNYSTNWYKY
PPQARRMLVLLIAGTTRNFEIKAGPIFIMTFETCSSIFQSSMSYIATFKTMYTAF
Q

>TpreOR60
MDEIEKRYETLSKESLRFLGLWPTQEKSKRYKGFALVAFFMIKLTIPQFLCIIVNPDDKFIFFENTMILTFYIMVSSKHG
AAMVNFSKVKALMLSIDELKKPFTDPHELELMKKYEGQGFLLARIYAIIMLITVLVYNMVPFVPPFLDVIYPLKSGPRP
IILPYRGEFIWFKQEDYHYYVSFVAIFDVLIFLPVFVGIESVYTTTVREICGLFEIVCIRFQRQFSRAEGSPTSEISDAVKLY
NECVRCVQFLEGTYSITIFTTQITSLLIFSLCAIYILYVNNDVFNGLRFSTFGIAILIHMGYYFGIGQLLINSSDKVHFSIYSS
NWFLASAKLQRLLVMASTRTLYPCFITSGKLNPLNLESFGTILKRIASTISVMLAVL

>TpreOR61
MYNSWEEYLLCQCLLLLLLLGYYLKKTDFDYKCMVSESEENAHHFLTQSFKYFIQQHQRCIKLYEILYFLTERLMFVIM
LGSSALLIIAGSTMVVLMDSHVPMAIKMFVCCWAAMIVILFICVPGQLLSNAGHEFFIECYCVNWHGYPPKARVLL
LLILVRTKKPLVLRAGFLADLSFETCSSVLKKAASFVTALRSIYTAF

>TpreOR62
MIIQSVDLGFHEFPMQFFIFTACGVWCPSDWSPSLKKLYKIFEIFQIIAATWFWLTMFMNLLLKNTESDLFYDNIFAL
LVLTLAAYKKYFLLKNRKVISKVLSMCSDDSWYQPKNKQEAEIIERFENETRLVTELYALGISTALAIKAVIPMLKSKSG
LVLPIEVWYPYKTENIFIYLFSYLQQLIAGIPMICLQISVDSFFVCLVLRVVTQLNLLKYRMQFSSNNFISKGQEVTTAT
GIHKNIPMIDTWLRICSRKHAKIYEFAESVHKCFKSIMAAQLIITIPCLCMTVFILAQFDELGFNFVDKFFCFATCLMQI
FLYCWYGNEIILESLDVENSIYHMNWIALQSKTRKNILMMMLRARRPIKFTVRSYVVNVASFIEIIKTSFSAFRLLQT
MS

>TpreOR63
MLIKNSLEHQVLPVPFHLLTLWGVWCPENFNPSIKKVYSVFTIIVLAVQMSLTLGVSILFVMMISSKNFDLDIFFMM
TSLINGMYKALNIFYYRKRILSLLTRGFEDRWHVPRDDYEKYILEIYFNESWRTHLIYAVACLAGVFIKLIGPMVKYDT
DLKLPTPSWYPYNTNETVYFWVSYSQQMFVGGSIISMHIGADTLLSGMILQSCVQLQLLKHRLRSFSKHCIDAHRYL
GTKLSKSSIESVFLRQYICDHQLIYTYLKMINHDFSGWLLAMLAVVVPNICINVYLLSFQKIGMNVDFVTTFGLLSISLF
QIYLPCWYGNGVILHSTEITNAIFEMDWTDLSPFTRKALIIMMMRSLKPSKIETAHIIPINIKALLKIMKTSYSFLSVLQ
QM

>TpreOR64
MDELARMFEAKETWRYIILIEKLAGCWPYQYPRVNSIVRVFNGVMLVSLLVPIVCRLLIELNCVDMDIQLVIENTFVS
TILSGVLIKFIALYINEKQREEVYQKMTFKTLKITNNKETQVMNYHSRAAPLFLTKILLCVGLTTMMFETIPLANMNLE
YYLQSNLTFKIKQLPMYAEYFVDQEKYFYALLFHMFVSSWVAVFINISFDAAFVLIVHNHLALYKIVSMRLREASKND
DDNVCYCKTISYEHETRGNIINAIEIHQTAIEITNTIEKAFSVPLFLLTVQNMSTFGGLLVLVMTTVRDPVELFRYFVTF
AGVLSYFSLFAVLGEKVITNSTAIFSESYTTNWKNHSKPLTALFRIIMMRSMKPVDIKGGSLVLGMQTLGQFFQQSF
LYILFFRLFRRPA

>TpreOR65
MIIDTLNELFTLMTIAGAWRPLDQLSVKYRIYTVTRFVLLPLPFIIITGMILGLILNNSDKDEMYTTLLLMLTVENNTIR
AVGLWMNRKHLVQLLGMLASPYAQPRGKSEIEIEKSYSEFLRKFLNLIYVWVGGTWLIWVSPPFFQKKDHRNLPLT
VWLPHEGPIPDRIFWWLWIPDAASFLISIILIIGHDISIATVMSFVSCQFDLLAHRVRCMTMEAKRRAHRSSRSIEAC
EKEVIVENVNHHRYILEFADLLSKGYGCSMIFQFSNCFIQLTANTYLLASTTTIDVQLLMRLIFLSCMFLQSSIYCYFGD
HLTNKSRELTYALFTANWMDLCLRSKKDILFMTARTMYPVYIGKAFFIVLSLNSFVQILRISYGVFNVLRQT

>TpreOR66
MNFFQVFVVKFIAGMFEMLGRDMARIHEEIESSSAFMMNKQKIYKIINRKIRDNIRMHQEAIELFVGLREVSQKKF
LIIIVILILELCSTGIWFVIVIPYNKFYAAKMAFSMVTVVMILLYLIYSSEDIIYACNQLNSMCYDTKWYLLSVKEKRLILF
MILRTENPCTLMAGPTIFMNYETFSAILRFTLSYLMAFYRIMEQSSYLN

>TpreOR67
MSSKVKKVWKNEDGYLRAERMTRSILLSVGLWPLQKDYPRFLRNFLVAICVALSLFMMIPLGIFIYSDAPNISVRLQL
SAPLLFSALCMSKYANLMWKGPKIESCLRRMAEDWQNVKDPLERSTMLDYARRARSMTKFCMALTFTGGLFYST
VGPLSRAPIVVDNVTLHQLAYPGNFIFFNPRTRPAYDYVFALQSLGSVVRFSSTCGVCSIFIWFVMHISGRIDVLGSTI
ERAVDQLDNRMLKRIVDDQLNLYRLAKELGDIFNELCLVEFVGNTVLICLVGYYMIIALLQHNYTRAYTLAISLSSMT
YNLFILCYYGQILMDKFDELGRSIYMADWHKLSGSNARSIVLLLVRANRPFALTAGKIIVLSMASFAKVIKTSATYFNV
LWNITSHSMNAQ

>TpreOR68
MMSDTMENKELEAGIKMCRIEMEILGAWPDTKYNQWYLKFRYLVPIFLSVFFINIPQTRMLIIVKNNLDDLLEILTTA
DLIVFIALIKFSSILAKRKDLMVLLNKMRIDWNTADRNHKYEMRKSLIFSKLLMSCYNVCTSGTIIIYAASRFLVLRQH
NEETRCSNTTSMKPMFIKSKFFFETEYSPMFEIIWIGQFIAAFVAKMAFVTYDGFFIFSILHLSAQLINLKIDFRSLPVEE
SGQNFTQSLKCLVQKHWDLKKFRTLVEENFNQVFLVQMMCYSVTLCLQSYQIVTILTGESEKNSATLEFIVIFTISNIL
SLFMYCYMGEKLSTESSGLHYAVYEIEWYRLKPSESKLLMIVMHGTKKPLVITAGKFTNLSLSYFMQILKTASGYLSM
LLAVQDRL

>TpreOR69
MTEATKSIPIENTWDFTTQAELSELSRQLDFEENHFYWLKKFLKFVFLCPVQPKIIQKSGRAMMHSTFIFSITVTCTK
AYKELFVNEIYPPMLAELAFQLIVLSTTYIIYLQAIGMEKEVRIRIQSLKNPNDPNRMCEIITIDWLGINDPEEKDIMRV
VCERGSRLIFIHFGVLLPCFMGYAFAPVALPYILNRYLPENKTLKRSLCVHVEVFIDQDKYFNFILCFVIHMIMLVLLITC
ALDLAYTSCITYIMGKIIWIGRVFEKLNEVNIYDQSPSLKQKANIYIHQTVIGLIKRHQKCLDFSQTLNDACSPKFIIVVA
SLVILLSLSGSMAVVELSYDVSSAAKMTVAFTVLLVIVLVMCYPSQLMLDASHDIYFKCYSSKWYEYPVRTRRLLILM
MIKAAEPCCMTIGPTTPLNFNTAGKIIKTALSYVTTLVSLCAL

>TpreOR70
MLLVQFQVSVLAYNSMYLVDGVNLIRDKRLNTEYVCQLMLIENICIRYLMFMLQRQNIARLMDKCQRLWTRLKSN
EVWSYVRPFERRVYFYRNFSLVISYLVIVLFVAGSQLTHLAPDRINGTARRKLPYSYYYDVQEDPDFSIISGIQGLILCYT
AIILAGIDTIAPFLIMLACGYSVTLKNRLLNMAHKDDKSTKVNNQLIYGDVIECAKFHREIMSYCQDIENHMRSFHM
VIMICNVVYNMSLIGIQILQNIEYFFQYSSLLAMHFMQLYLSQWAPDHLLHETKAIGNAAYFATLGHSTYNHQANKIL

QIMMLRAHCKPVQLTAGGYIKLSMETFGKVNIFLFSIKTVEKFNAYTQFFSDDNQCGFYVYSCSKFYFVKIYILNIIFIF
LVIKQLEFHMHEII

>TpreOR71
MGFKLNQISNFSRKKDYLEKNDEIFKRIYELVQLQQFSIDCFETLNKFINPILVLGVLAVIIGCSASGCLMVMKFNEDL
NVAMKMGLIYSLCSEFLFVAAYPSQKIYDACNEIFDNCYCTDWYRYSIKNRKMIKLIMMRSSRHSCIRIGPSKILNYD
MSRKIIQLSLSYIVSLMSIYKL

>TpreOR72
MTRLLHKVARSWPPDEKNHRHGILRKFLHVSPPIICIVLYIYNLSGFMTARTVEDILEVINFVSVFTTATGRNFLLALTK
PKIHALVSKTIEMEKTRRADELSSIQCQIMKKWRNIHDKLTKFSIYGVWVGALMYLIPPIFFSKLPFPGYSTKGLEESK
WFYAIYFLQLLFACIFIPPWISVDFYVCTFLCTLCRELDMFYDAMQNIRDKDRSYLYRVIDRHSRILTYGMEVCDIVSY
SFGVVHASYGVFLIFGTIAMTQINWSTHGGLAIRNIITMIVCASSLSLMCFIGDLIQDLSTRLGDCIVLDNFLDRRKDR
KYLKLLEIVHARSCCSLKIKYSPNMIVNMQMYSVTLNYILSIYTFALTVMA

>TpreOR73
MVFCWFAISFNNLQCITIGFIIAHIHCLRYYLNEIDIEFEQNEKRRNISEQKEVDRKLIQLIHQHQSCIKLYENLDIMGK
PLFFCSITTITFNLVCCGTAMVILLDSDFSGAAKMFIIYSCSIISFMLLCVPSQLLSNASYEFYKESNCINFHKYPPKTRVI
LLIMMMKISDTFVLRAGPIYELSFETFSVVISSFQLQLKSQN

>TpreOR74
MQSISSLLFTKRIKVREKNMKSMGCWVPSGTFVFEYLRVNEKLYRYNFLWPDQSTTLKFFGRSLFLLSLLTYQIPQF
MHSAINYQQQKITIHSAIENLLGTFLTFNLFIRYVLHMQHQETFKYIHVVLTKHLSDCDNYQEKLVMERIFSSKTRHL
QKIMIFFWTLLSMFILVSAPIPALINAITKKETLTKQLCFPAEYFVDFDSYFWLLYIHQLICIFFQCLTANAFDITYANNA
HYIAAMFGVISYRLNRLNRFTRNPQLTSRQRDEYIGRELLEIIDKHNRVIDVTAKINRAYAPMKMFSLMAMLMLIGE
VGFMLAIDFGNVMTHMRFFIALAGLLLYLRFICWPGQTLIDASLSVFYSSYMNDWYLFTPKLRYLVRGIMWRASKP
CVITAGPFAVISSETFYWVIKSSYSYMSFLMKMDGESF

>TpreOR75
MLCTSGELYPNREDYYYFWLAYTTFLMFSIASIVVAQISLCIYFVSINLSKFEVVCYHLERLREMSDQSDENDPNEIEKI
VKHTCANHYETLNFFRLFQIFSRNVLAFLSTYVFVALSTTGTMILYAFKIKNTFLILEMIVSFLCILCFAFLMGHIGQILC
NASDNLFYQSYFCGWYNLPKESKKILHMMMTRCLTPCELQSAPLSFMKFNYENFNAVVNTSISYMTVIASVM

>TpreOR76
MLEDQKTENEFFDSPEYHLNKKFMTVVGGWPYLSPKKRKTIWFVVNTFVVTVWVSELIHIIVIIDRTKEVIYCLMAFL
CTFLAICGSINAAVQPRSNKRLVDSLRDNWNNLRSDDERKIFATYAHYGKILSSVLAVCYYLVGMTYIVSPLIPSALHY
RITGDWIAPTRNVLEVEFFIDPVKYYWQLYVLLVHMCMGVVSLLLANDTFFFMVVLHCCGMFAVLRYKVEQMDK
EITFNGYSYNQQLAVKRIEEIVMYHLECLEFANKIEDFFCIQFIVQILVNTIIISVCGSQIIQLADQSLRDSLLFAFLTSATI
FRLTFFNVCGQGVYDHSLRVHDQLIHTTWYECPIKVRKLFIILYNRSAEPYNLTAAKMINLNFVTCTQLLKTAMSYF
MMIIQTP

>TpreOR77
MNRKSIDLSLNEAGYEYAIGYTRNLFLPFGIWPVRDYEESRGASRARAIANFLCLFILFFVVGPYFVQTFLIEKDNQVR
IKSTGACVFGITNVAKYLVFLLRGHRIGSCLAEMHLDWRSITNDPQRRLMLRNARAARLLTCCSFVFMFGGGVPYV
TVLPLSQPPLLSADNRTLLRHLSYPSYFGFFEPRVRPVYDLVFSVHFCFGILAFSLTTGLCSFIAMCTLHVASRCAFVSA
MYRELGRNFDRELLSRVVLQHRRIIA
>TpreOR78
MYMLENQCKSKTYLEHDEELRKAREGCDVVEECQLRNLEFLNRERCYVFRCRVDGRANLKRLELSCVPQVKIVLIAL
SGLINKNVPGHELVEATVYSGFLLCVLSIYALSFDDKLIAASSIMMEIWKEFREEDERSIIIEKLRQGFTISMLYSVIMYTI

FVGCSLQPEVKYRAISWISVNKTIEKDLFFPIYFTKQLKDNYYLALFVFVLGGLMLGTILSIFNYFQTTLWKFIVASLLLI
GLLNVLQLFAQQKYFIVIIVIIIELTLAGFWFLYVISYDKNYALKTVVWMIVAICNLFYIIHPGQELINANNRLWGGCCN
CKWYIFPAKERQLILLMMIRTSKPFLLTAGPIIPMSYETSAR

>TpreOR79
MELYDSRYFVINKKLQKLIGVWPFESRTRKNFCRTFGFIFMFIGVIPQVIALDVARRNKDFDKITQSFATFLFVLAIYSK
LVTSIMKEEQMIKLYEEIVNNWNQMKEKDDKKEMIKHAEIGRFMTICYAAYVFNALIGFITFPLLPSIMDKISPLANG
SRPRIYVLEGQYMVDREQYFGIIYILEAGVCIMMVVIFCTCDTAFCVCVEQSVGLMSVVKMRLKKATKYGSQWEKT
DPNNTPYALVKKLIVFHQKVLTSVEIVESAYSFYLFALMGFNVFILSCGSLVIVANLDNPMETLRYGMIFIGLMIHMF
FLNLPGQRLLDASSDLHCNAYDNEWYECSEQTKQLLLFIMLKCKTPCIITAGKIVIMNIQNFGILAKSAASYFTVFASF
R

>TpreOR80
MDFEQLKSWNWVKFLEINAGCWPFQNPRKNLYIRVFSFMAIVTVYIPLAIKLMLSLDQYQEIVIENSLFLMIIHGLFA
KSIVLYLNEKQRGDLYREALSHLDAMDDEGEKEILRVSSESATKWMLTYLFTTITLGSFYHIIPLAHIHMNYFLRANAT
PPRQLLHINVEYFMEDEDIYYYIIGHFLIVFVFGGILFFAHDLGFMALVKDNCALYEITCYRLRNCSSRGSSDSRRSQGL
ADDEIHANVLRANDMYQLAMSITDRIEKAYNITWFVLLVQNMSSFGTCIMLLYIQEDPIEFIRYITVVGSGLIHFQTIF
YSGQKIIDSSQAIYHACFHTDWFTFPRRTRNLLIIMMMRSAKPCKLTGGKAFILCMSTMRQFVLKGLVYFLVFAVE

>TpreOR81
MYIMYYLKYGLILLDSWPGVDSAKLCNALVALSCASLCFQFWDAAAVFHDLDALLTNMETSIGVLSSVFKIVTFRM
QSASTKKMVKMSIEEHVEKNAANPNSKTGKSKSNNENAVKLILQILFVSYFVLGLSYPAVSLVSYALGSSEERVFVLP
SMYFIPSPRESPAFELLWIYQFVVVLFSVLGQCIADSSLIILNLSIENDLRECSFYNFQEKEQNLVRYMLVRAQNSNTL
EIGKFGNLSLFSLTMVKLDYFNA

>TpreOR82
MNPEEAFSVYRSFLWFLGVWPLEEKSRYQRLRYYTAAFFQASFLLHTSIEICLNRSGVSDMVDVCLFFASAFLALIKH
TYLHLHGRKIAYNLRSYMRDWRQPSEYGSAVMRSHFKMYRYQFIIYNSIGYIGTTLFLIRTLLLNYLKDRGQARDDG
PEFEYEFICKISFLSKPFLLRYHTGLLVLQYLQCMYVCSSGASTDCFFFGLLLHLAAQFKILNRRWHDFGDASRDDQAS
FDELVARHQLLTKLGQHLEQSFSRVVLLQLLISVILICMSGCSILVSMMERDYVTMLISTNCVSFMITESFIYGYASDYL
KSQSLALVDAVAACGWYDLERARRRDLAFVLMRALLPCSITAGKFFYVTHNTIVQLVKTSVSYLSVLRMTIEHSRKA
DAN

>TpreOR83
MSGFYIKATWSYRVLSFLGKSLTIWPLDRGQSKGLGLLLNCFWWFYLLNYVAILVPTLYGLYINRRNIVSASYSWIES
TVFTEATAVMIFSRWQRSRLESLFRLAEKQLAVKKRRVVRFYANYYAIVYLATLAFYVCVVMMYIILEKPKTGNNELA
LSASYPFRLEDNPIKWLLWVNQAIVFVHAYIVANFDGIAVFLIFTCTDRLKQLDKHFRDSRSYEHLVACVREHNDVLA
LIKDTNRILRFMVLKTTAFFMSYVFGAGLQILNNTAQTVMIHQISILFLAYARLYLCAESANNMSAAGRDIAMTVYS
TSWYDETPKMSTAKTIIIQKCQREPVICVSGLMSALDRRYLRGIFSATISYLMTLRTIVGSK

>TpreOR84
MATQTTRAELAELSRQLGFEENHFYWLRIFLKFVFLCPVQPIVIKKFGRFIMHASFLFSIAITCARAYKEFYINEIYPPM
LAELTFQLITLVGVWCVYLQTIYSENLLNKICEFVTIDWLGIKDAEEKKIMKVACERGSRLIRIHFGFLLPSLLGYALVPV
ALPYVLNPYLPENMTLERRLCAHVELFVDQDKYFYHILIFLIHMVILIMLIVSALDLAYTSCIAYVMGKIIWIGDVFEKL
GGIKISDQSPSSKRKMNVYVHQTVIGLIKRHQKCLEFSQMLNDICSPKFFISLVVLLNLLSLSGSMAVVEISYDASSAA
KMAVAFVMILIMVLVISYPSQLLIDASNDIYFKCYTSKWYEYPVRTRRLLILMMTRAAEPCYMTIGPTVPLHFETASTI
INTAMSYVTTLVSLCAL

>TpreOR85

MESVGDVVRVKEESKDTWPDAGDDQNFDSLDFDNFEARNVETFLGYKSSMVVLMDSDAVMAIRMLVISCATLT
VMLFMCVPGQFLSDASNELFTECYCVDWHGYHPKARMLLALILVRTMKPFILRAGFLADMSFETFSSIIKVTENTKE
YGFSSSLFIEDLFQVIASLMMIYVYPFMIWKYDRSYEKSNRFSLVITMLFIPITVTFAFAPLVIPILSTQFLPGNQTFQKA
LPLHVEFFVDEEKYFYHLFALQFITIMIYFTIIGGLNTFLYSGVGFIVGELQYLQYYLEKTDSCYSDMVSECRDIAYNFLT
KSLVYFIQQHQRCIKLYEILYFVTESMMFVTMLGTSTSLIIACSAMVVLMDSDESMASKMLVLCIASLSVILFICVPGQ
FLSDAGNELFTKCYCVDWHGYPPKARILLVLILVRTMKPMELKAGFLAVLSFETFSSVL
KKSMSFVTAFRSIYATL

>TpreOR86
MTKDPRASDIQHFEWAFGLNRFVFGLMGVWPKKRPNDDSNSNSSFMSTNVLVIPGMIVLLVGGLIAPQMYALSR
IYDDFTLVIDNLTTVNPCVCAVMMLYFLWSNRDSVARITKIIELDWLRDVQRSPGERLTMLRYAGYGRTFTLSGCFI
TIFAVLCFVVTPFLGLSFRLINNRTDQVGRRRIHLPLQSVYPGDYLRSPYYELCYAAQMLGGCIVGMTIAATDNFFAA
LTFHASARCRVLAERMAGLALIGIAREEEFFRALGASIREHVRIIRLVRTIERIFNQLLLTKLICMPLVVCFVGLELIGSFG
SESIRLTTLFAQVGALVTMVFHALIDCVACEVLMKYEHQPSRLRFRVVRFTRALCEMLHSDSHTAQVSAETYGW

>TpreOR87
MSEKNQEELGLFDTEYWTITVKLQKVIGLYCFQSDRRNCISWIYVFVFTLSFMISMGVRLINEIGVHIEIVVENIVGE
MYLLAVFNKLVISVLKRKSFKKFYAKVADHWRMIEDAEELKIMTDNMRNGRDVVKLYSIFIIIGTTIFLSMPIFSPLLD
YVVPLKNATRPKALPYYAEYGVDIEEYYYPLIAQAVFGGVGTITVLVTFDLGFMMLSHYVIGLFALAKYRLSKVNTLM
RKIEERNGNPWKSNWPIPYILEAVNVHRQALAYVAELEDCFNIAWFITLILNMMMFGGGMAILLLKNNPEDMFRY
TMVLLAGFMHFYYIFLPGQEIINASEEVFDVCCACGWYNLSEKSKFLVQFIMVRSLQYSFLTGGKMFPLSMETYCN
VSKISKG

>TpreOR88
MTSAMLFYVIMPVAPTFFENVFSRMNETKSFDFVMKGEFPVNNMRDYYFEIFIFDMLVCVATVFVLGAVDSTYAA
CTEHCIGLFGLLKFRLVNLTPKISQNYSDMANSNYDQDDATYCINRNYTLLLSFFPRFAEILKTSYSLTFLILMGATVIYS
SLVCTLILLKGDELKDRLRYSGIFVGLLIHLFYISWPGQKLIDHSTGLFDDAYTNKWYECNFRTKNLLRIVRLRCLTPCQ
LTASDLYIMNFPNFAAVLKTSMSYMTVLASFI

>TpreOR89
MLRDQSCERDTFFNGPSYHLNRKGLILCGGWPYLNPRKRKIIWWTINLAIFTAWVPELIYIIEIFDDAQKVIYCTMAF
LISYLAFCASVNAVVNHNSMKQVLDSLKDNWNDLQNDEEREIFEKYANFGKIFSIGLSVCYFGNLSLYFVSPLIPSML
HYRATGNWTTPEKNFLEVEYFVDPVKYYWHIYVHGAQAGSWVVCVLTAYDSFFIVLVQHCCGMFAVLGYKIGKM
DKEIHDYRHDQHSVVEKIEKIVMYHLHCLKFAKKIEDAFCMQMIQQIMINTIIISVCGSQAIKLADESKQESFRFAYLA
CSTIFRLTIFNILGQSVYDQSLRNIHQLVRVSDGDEEIIHNPLQQKRRALQSHGCQDNLPESYYLFEANENGDVLFHD
DYPNVMRCLFLMIKAQAIIIRH

>TpreOR90
MGLSFGCFLSLRVHFHYLCRNKMSPNEVFDNKYFRHNRTLLKQVALWPYASTSSKVVKRIFIIVCFYSMSLPQAIRGI
EEIVSVNVNQEIVIENLTGFLYFHAVIAKVITQIVTEKRLKYLYEEISNDWKIITDKREKAVLEKSAAVGRNLTLAYTGFV
VISAILFISINAFVPVLLNYILPGNTTYQKQLCIYAEYFVDQEKYFYYIFTHTMIIGVMTVYVATTIDCVFVCCVQHVVG
LFNIIKYRLREISRIYDTAVNESIIDLHFDAKKYLINVVEIHKKTLDLAELIQNTYNECFFLLTILIVAGLATFTYVLAENVH
NPLNFMRIWCLWFGAIVYMFFVNLPGQKLLNISEELFLALYNSSWHKFPMKTRFIIQVMMIRCLSPCRLTAGPLIEI
NFQSCSNVNVSSIKYLL

>TpreOR91
MLFEICDEREQTLLVDIRDQLGLTLLQWAVAYHLPNAILWVLKEKKEMDIFNNFMVEVIFQLLASLGLMFAYVSIGA
IKNKIQLVVARMWYDVTEDIQNIILVLVSKVVYIATIQAVLLITSCVIYALAPVTIPFFMTFISSGNQTFKIALPLHHEFLI
DKDKYFYLIFTEQLLLLTDFGILVAGMNFFYCSHLVTIIGGFRGLEHLLKKLDDEYCYKISVGDKNANEWFNLQLKDFI
RYHQAIIDFYKISNEVSSPIMFFLLLGSGCSIILALTSIVVATDTAMAATAKMVVGTLIAVILIIFICFPSQLLKNASEDLLL
ASYKLNYQNYPP

>TpreOR92
MVCKEKIHTCALLMGRMCDTNAWETQAIMFKPLKKGYRISIFQAVAMVILCTSFGISPIAIPSFLNRVLSGNQTHEI
NLPLHTEMLLSEDEYFYQIFAAQLTSLFIYGLLISAINALQCLSVAYIIGEIRILEYLLHKMYLEYKFKNLVDEKKRNKCIYE
QLKYFVDTHRICIEFFNIVHDISNILIFGILIGATCIVVLTATSMIILMETDLTGSTKMLFAFCVTMCAITFVCFPSQMLK
NASDDLMFTCYKMNRQSYPAKIRKMLLFIMVRTVKPFVLTAGPTIELNLETCSTYYRLKFKIAYKETFTPSGPGSLPK
NVWGLQEPNNKAPYAKRGFLFY

>TpreOR93
MSEEIKLNEVDDFFDLHYFALNKKFQIFCGLWPLETGYMKYFKQGAMGAIILANLILFSHALGTFCGTNMDYCCEN
MLGVVYCITGFSKLVGISTTGGKFTGIYKMIARNWRETTDEIEYSILEKYARISKMLTRLYIVAFLMTGGVVTQAPAIP
LLLNYIIPLNESRQAIPIVNTDYSITPYTRFAHLWVHYSFTAVAVACVFIAIDATFVLIVFQILATLDVVKQRIRQASSVE
SENAEQSYGILVKAVKLHKDAIEFLNLSNEANSLQFLVVLGGTIFNISFGSLAILSRAGAYADFARIGILTLGYLFQLFVL
CLLGELIISSSSELFVIP

>TpreOR94
MDKRLENLKPGRLFSMESNIYYNFHLLRLMCIDPYMKESLRLPVNIIILTTYLLTILMTIVVLVKAIIKNETNINLIMEMS
FTCIFTVDMMSYYIILLKYNKEEKYFYLLLAIVIVINALMIVESVLLLSANIMIIKYLEGLFDFMSYSVNHMYDNLNPIMI
KMSIKTSNKEIRRNIINLIKCYQQSIQIFNLFSTFTRYNYFIVVPQIALNIIFLGSKALLEISHDLQLFGRLIIICLASFLVLPV
LIYPCQQLINANERFRNECYACKWYKFSPEARRLLLGLTFRASHYFVLKAGPIISMTMETCSTILKTSLTYLVTVNKIYS
SFGEK

>TpreOR95
MAFHSKWTEKEQKFKNYILHVIAQSQHPVTIKANNLLPTLSLSFFSQYISIWPYDLTADKREIFIHQIRWWISFINVCIL
LVPLILGVYYFRHDNVKMTKTLSELTCLTEIFFNLIQSKLELKNFQIVFHEIGTFIKEANKNDTALLQKYLNKYRDFQLL
MGLTFILVAILFSLMPIVTRQSLPADAWYPFEIKSSIILICIYATQVLAIFQTAFGIFVDIMVAFMLWFSAARFEMLEIEL
QKAVSELELKNCVHQHRKLIILTNKIKTAVKLIILKTNATMLLAVICGAFQLLHHESLEVLIQFVMLVAAGCLRLYVSAK
AADDLKENNDRFSRSILYTPVIRKSKSASKLSSLLTFFSQRPVVVSIPGVIKAYTLQYYASFLSTTVTYFLHLRIILDE

>TpreOR96
MNELKLTREFDNHFFLTKICMIYIGLWPLDGACNDLKRNLKVFTGYFFVVSLILVELYDIYFYSSGSIEATAEIVITMTY
AFGGLMKISHFLKEFGVFKDMWHTMHVDWTGVLGNDQERSYRSHVMRRITIKTRKYCRQYALLTLGAAVMYMT
MPFIGNQAHRVRKYPFFGRYYYDDQSDLVYILCYFSQVITGTFCATTNYALDTLFLICSYHMCAQLKILKHDLIKMSK
ENVAQRLTCLIRRHQREIRNVKKLQKVFSNVGFWQLFVACVITCINIFKMLNSQALGDAIYKSGWLDFQGNYKRDL
KFMIHRCQLPLTITAGKIYTLSIANFMEVIVNYIICQLSNQTCRL

>TpreOR97
MSSSVLEAKLERFIDISLKSFIVMRERNRKHQNNLKLFETEQWKITVGLQKISGLYCFQRDRINFISWLYTFLFTSSFM
TALGIRLVMEIGVNTEIVIENFLAELFMIVIIFKLGISVLQRKSFKSMYTKITDHWRMINNVEELKIMTKNMNNVHVIA
KVYALWCFFGMEFFLAMPVGYPLLDYVIPMKNRTRVVAFPCYVEYGLDPEKYYYPLMTQGFFGSLGCVAVFAAFD
LSYMMLTSYVIGLFALAKHRISKVNAIMQVMELRNVNALKSNWSIPYIVQAIEAHQLALAYVNDLEDNYNKAWFV
SLVINVTTIGGAYVVILVKDTPEEIFRYSAMLIGAFVHFYFIFLPGQKIINASEEVFDACYAVNWYKLSNKSKYLLKVIM
TRSLRASYLTGGKMFSLSMDTYCRMLKTSLSCVTLLKRILEF

>TpreOR98
MMKDAEELVIMSKNMNQGHRIVKIYSLYCIVGMGCFIAMPIAYPLLDYVIPMENITRTMREPVYVEYGIDIEKYYFP
LMTQGVFGGLVCVSLFVAFDVSYMMLTNYVIGLFTLAKHRLRKINRLIWVMQQRNVNTKKSNWPIPYIVQAIETH
QLAVAYANDLEAKYNKAWFAPLLINITSIGGCFAILTVKDTLEEIFRYFAMLCGAFSHFYFIFLPGQLIINASEEVFHAC
YAVNWYNLSVKSKYLLKIIMIRSLRASYLTGGKMFSLSMETYCSMLKTSFSLIAILKRLM

>TpreOR99

MSERNRKQQNRLGLFQTEQWKITVGLQKISGLYCFQRDRINFICWLIIALFSLSFTIVLGIRLVMEIGVHNEIVIENILG
ELYMLIIFSKLGISVLKRKSFKCIYAKIADHWSMINNAEELDIVSRNMERGHGVVKIYSLYCIIGMGCFIAMPITYPLLD
YVIPMENKTRTMGEPAYVEYGLDPEKYYYPLMTQGFVGGLGCIAVFVAFDVSYMMLTNYVIGLFALAKHRLGKVN
KLIKAMEQQNINVLKSNWPMPYIVQAIKTHQRALAYANDLEANYNKAWFITLVCDTATIAGCFAILKVKDTPEEIFR
FFSLLFGAFIHFYFIFLPGQLMINASEEVFDACYAANWYKLSHKSKYLLKIIMIRSLRASYLTGGKMFSLSMNTYCSVY
AKDQLIVRRSSEEIVGFLKNILFFKFHV

>TpreOR100
MLRDQNLETDKFFNGPLYHLNRKSLILVGGWPYLNPRKREIIWCTLNLAIFTAWVPELIYIIEIFDDTQKVIYCTMAFLI
SYLAFCASVNAAVNHNSMKQVLDSLKDNWNDLQNDEEREIFEKYANFGKIFSIGLSACYTGNLSLYFVSPLIPSMLH
FRATGNWTTPERNFVEVEYFVDPVKYYWHIYVHGAQAGTWVVCLLMAYDSLFIVLVQHCCGMFAVLGYVIKLTLE
NSYTIGMMDKEVHDHRHDQQLVVEKIEKIVMYHLHCLKFAKKIENSFCMQMIQQIMINTIIISVCGSQAIKLADESL
QESLRYSFLAGTAIFRLTIFNILGQSVYDQSLRVHDYLMYTTWYEYPMETRKSFIILYKRSAKPCNLTAAKIVCLNLITH
TKLMKTAMTYFMMIIQTS

>TpreOR101
MYIYPFLHWNYDKVILVVVTMKRILEENDALGRIIVKNSYEKSNAYSAYMSTLFIFMGTGFSIGPVAIPIFLTWIQTSN
ETHEKSLPIHTEFFIDEDKYFYQLYAYQIVVILLYIILMGALSSFLFSAIGFVIGELHYLQNVLEESDVKYKSKLRANKLLA
HNFIVNKFKFVTHQHQNCITLYKNLNNITNSIMFVVIFGSSCIMVLSGSTMVILMSSDIGMAFKMSIAYFGSMIIITM
ICVPSQLLSNSSDSLFMKSYSLSWDEYPPKARRTLVMILVRTIKPLALTAGSITELNLETCSSIIKSSLSFVAGFRSLYTTV

>TpreOR102
MSNSHHQQQSVSNHHHHHHHHHHHHHQQLQQSTLFYLSFPVIDQLARSKKNKAMDDIERRYEKISKFSLKSLGLW
PTQSKLKRYTGFTIFTLLVVKIVYPQIVNVLKHSDDTIIILETAVYVLFHIISLSKYVSTLLNLSKVKALMLSIYEVRKNLTD
HHELKLMKKYENEAYFLVRFYALYVLTGMLLHSLVPFAPPLLDAVYPLNNGTRGRLLPYRGDFVWFKQEDYHYEICV
DFIVSAMIFWLLYAGIESIYTSTIKQICGLFAVVCYRFERKRKISSNVDRLSKQNFDEDYGLTSAEIADAVKLYNECIKCV
GFLEDTYSIPVLVTQISLLLDMSIVLFINEELLNGLRFGFFGSGMVLHLWYYFWIGQLVIDSSSRVHFSV

>TpreOR103
MDALVDKQIKKDMFWSRFFLQSACLWPYTNRILKYAGRVLLIPSAIFMELCLLYGGYKNLRQDSLDIVYTFTNVIELLI
GISIIPMYFITMSYDREFKLLLGDMMHFYSEEASAEERKIVQDVTRGTYHLSCLLAAFVCVFCAAYALAPHSVWYHC
APVLLVPLLRHCLGHYRHHGNHSSGSSRALASTAVVFAVDQDKYYYPLFVYTTMNMIQLATVANALSASFLCSVAFI
RAQFRIIVHRLTNLDSLVQREYLKRDLSIQDYIHQEFINLYLYHINSIRNLKLINMIGEKLLFIVSVLCLLGLSFSGSMAVI
FLRKAPLAALNFVMCFIVLLILSLMLNYVGQSVIDANNEVYHNCYHCGWYGFPVKTRPFVFLMLMITRKACGLKSG
PFTILQLSYENYSLILKSVISYITVMISVL

>TpreOR104
MTTIFQDNDTLGQKVILRSVKKFMTINSIITATFVYVSVNGATAPVVIPELLTWLLPGNKTFKKSLPIYWDFFMDQHE
YFYQIFVMQQILLILFSFTVTSLIQYEYLCTGFIIGEFHQLQVFEQLYKLNSPVMFVVIVGASCCIILSGTTMVVLMNSS
SAIAIKMLIVYCTALITVPIACIPSQLLSNACLDMFNKTYCINWYEYPPTARRILVLFLLRSMKTMVFEAGKIIELNFETC
SNVLSYFINWYKYPPKARLIVVLLLLRSMKTLVVEAGSIIQLNFETCSNISLLI

>TpreORco
MMKMKQQGLVADLLPNIRVMQFSGHFMFNYYNEGLKFPHRVFCIVSFLLILVQYSMMGINLAMEVGDPDDMA
ANTITMLFFIHPIVKTIYFAARSKTFYKTLGIWNNPNTHPLFAESDAHYHSVAVQKMRKLATFVGAATIFTLFAWTTI
TFFEDSVKTVVDKETNETTIIPIPRLPIRAWYPFNSMKGFGHIMAFVYQFYYLAMCLTLSISVDVLFCSWLIFACEQIM
HLKAIMKPLMELSATLDTVVPNSGDLFKAGSDDHLRDTNGVQPAGDGIVDSDVRGIYSNRQDFTATFRPTAGTNF
NGNVGPNGLTKKQEMLVRSAIKYWVERHKHVVKLVTAIGDTYGVALLLHMLATTITLTLLAYQATKINGVNPYGAT
VIGYLLYTLGQVFHFCIFGNRLIEESSSVMEAAYSCHWYDGSEEAKTFVQIVCQQCQKAMSISGAKFFTVSLDLFASV
LGAVVTYFMVLVQLK
