# Supplementary material for: Olfactory Sensilla and Olfactory Genes in the Parasitoid Wasp Trichogramma pretiosum Riley (Hymenoptera: Trichogrammatidae)
Source: Insects. 2021 Nov 5;12(11):998. doi: 10.3390/insects12110998 (PMC8620382; doi:10.3390/insects12110998)
Supplement: Supplementary file 1 [file insects-12-00998-s001.zip › insects-1433424-Table S1.pdf]

Table S1. TpreOBP Primers designed for qRT-PCR.

Oligo	Sequences (5’-3’)	Oligo	Sequences (5’-3’)	Oligo	Sequences (5’-3’)
TpreOBP1F	CCAATAAGTTCCACGTGCCA	**TpreOBP9F**	TAGCTGGAGTCCGGGAAAAG	**TpreOBP17F**	TGAACGAACACGGAACAACC
TpreOBP1R	CTAGGCGCCAATTCTTCGAC	**TpreOBP9R**	ATCCATGTGACCGTCGTGAT	**TpreOBP17R**	TCTCAGGAAGGAAACCGACC
TpreOBP2F	TCAACGCCTGCCAAATTCAG	**TpreOBP10F**	GAACTTGGTCGCGGGAATAC	**TpreOBP18F**	GAACATCAAGAAGGGCCAGC
TpreOBP2R	CACTCGTTGATGGCGTTCTG	**TpreOBP10R**	TGGTCCAACAGATCGAACGA	**TpreOBP18R**	TCTTGCAGCTGTTGATCACG
TpreOBP3F	TGAGGAATTGGAAGCTGCTG	**TpreOBP11F**	AAGCTTCGGGAGTTCAAGGA	**TpreOBP19F**	AGTTGCAGATGTAGATCCAGTT
TpreOBP3R	ACCCGCTGCAATCTCATCTA	**TpreOBP11R**	TTCGTCGTCTTCACTTTGCC	**TpreOBP19R**	TGATTCATCATTGCTGGAACTGA
TpreOBP4F	TCGAAAGTGGGTTGTCTCCA	**TpreOBP12F**	ACCTTCGGTCTTGTTCTCGT	**TpreOBP20F**	CAGCAAGGACGATGACATGG
TpreOBP4R	CTCGTCGCCTTTTCCGTTAG	**TpreOBP12R**	TTGTCCCAGTCTACGTGTCC	**TpreOBP20R**	TCCCGTCCTTGGTCATGTAG
TpreOBP5F	TGAAAAGATTGGAGGGTCTCGA	**TpreOBP13F**	CTGATCAAGGCTTGCCTCAC	**TpreOBP21F**	GCCGCCAACTATCGAGATTC
TpreOBP5R	TGACGAAGAATTTCTCAGGGTTG	**TpreOBP13R**	TGCACGTATCGATGGCTTTG	**TpreOBP21R**	ATGAAGTCTCTCAGCTCGGG
TpreOBP6F	GCGAAAAGGCCGGAATACAT	**TpreOBP14F**	CTTTCTCGCCTGCATGTTCA	**TpreOBP22F**	TTTCCACCTGATCCAGTGCT
TpreOBP6R	TCGTCGACCAAACTGAAGGA	**TpreOBP14R**	TTTTCCAGTTTGCGCAGACA	**TpreOBP22R**	CTGCCGTCATTTCTCCGAAG
TpreOBP7F	CGCTCAAGTCCAACGATACC	**TpreOBP15F**	CAATGTCAAGGCTGGTGAGG	**TpreGAPDHF**	TCAACGGCAACAAGATCACC
TpreOBP7R	AGAGTCGTGAATGCATCGGA	**TpreOBP15R**	AGAGATCCAACGGCATCCAA	**TpreGAPDHR**	CTTTGTGGCTTGGGTCGTAC
TpreOBP8F	TGTATTGTCTGCACGGGTGA	**TpreOBP16F**	CGCAGATCAATTGAAGGAAGC		
TpreOBP8R	CGCTCCAGGGGAATCTACAA	**TpreOBP16R**	TCCCCGCTGCTTGATACTTC		
